# Nano-Carbon Biointerfaces in Biosensors for Cancer: A Scoping Review Mapping the Transition from Proof-of-Concept to Translational Applicability (2024–2026)

**DOI:** 10.3390/bios16070395

**Published:** 2026-07-21

**Authors:** Barbara R. Geraldino, Nilséia A. Barbosa, Priscila M. Galdino, Eduardo X. F. G. Migon, Danielle Godoy, Tatiana Cunha, Fernando M. Araújo-Moreira

**Affiliations:** 1 Nuclear Engineering Department, Military Institute of Engineering (IME), Praça General Tibúrcio 80, Urca, Rio de Janeiro 22290-270, RJ, Brazil; barbara.geraldino@ime.eb.br (B.R.G.); nilseia.barbosa@ime.eb.br (N.A.B.); priscilamgaldino27@gmail.com (P.M.G.); migon.eduardo@eb.mil.br (E.X.F.G.M.); danielle.godoy@ime.eb.br (D.G.); tcunharj@gmail.com (T.C.); 2 Brazilian Army Command and General Staff College (ECEME), Praça General Tibúrcio, 125, Urca, Rio de Janeiro 22290-270, RJ, Brazil; 3 PENSI Institute (PENSI), Avenida Angélica, São Paulo 01228-200, SP, Brazil; 4 Universidade do Estado do Rio de Janeiro (UERJ), Rua São Francisco Xavier, 524, Maracanã, Rio de Janeiro 20550-900, RJ, Brazil

**Keywords:** nano-carbon biosensors, cancer diagnostics, scoping review, translational readiness, biointerfaces, limit of detection, clinical translation, regulatory pathway, nanomaterials

## Abstract

Nano-carbon biointerfaces offer versatile platforms for cancer biomarker detection, but their progression from analytical proof-of-concept to clinically usable diagnostic evidence remains uneven. This scoping review maps 191 primary studies published from 2024 to 2026, covering nano-carbon families, surface chemistries, transduction architectures, biological matrices, and translational endpoints in cancer biosensing. The evidence space spans four nano-carbon dimensional classes: zero-dimensional carbon dots and quantum dots, one-dimensional carbon nanotubes, two-dimensional graphene-derived materials, and three-dimensional hybrid composites. Across these platforms, analytical sensitivity did not scale monotonically with nano-carbon dimensionality; instead, performance was shaped by the interaction between material architecture, biointerface chemistry, recognition strategy, transduction modality, and matrix context. A Translational Readiness Matrix showed that approximately 67% of studies remained at Low Evidence Level, whereas only approximately 10% reached Strong Evidence Level. Five recurring bottlenecks constrained translation: incomplete reproducibility reporting, limited Real-matrix Validation, scarce comparator-based clinical evidence, insufficient manufacturing-scale data, and weak regulatory or deployment planning. To address these gaps, this review proposes a nine-item minimum reporting checklist and a four-stage validation roadmap to support more reproducible, comparable, and clinically oriented nano-carbon biosensor development.

## 1. Introduction

Cancer imposes one of the heaviest premature mortality burdens in recent human history. The latest global estimate from the International Agency for Research on Cancer reports close to twenty million new cases and ten million deaths per year [1], with breast, lung, colorectal and prostate cancers concentrating the greatest share of that burden. The Brazilian projection [2] reproduces this distribution at the national scale. The evidence base on early diagnosis [3] converges on a conclusion that admits no ambiguity: outcomes improve substantially when detection is moved upstream into asymptomatic or minimally symptomatic windows. The biomedical bottleneck therefore does not lie in demonstrating that early diagnosis works, but in making it systematically viable in primary-care settings, with adequate analytical sensitivity, affordable cost, and tolerable operational complexity.

Biosensors address this bottleneck by a design principle that reference-standard methodologies do not possess: the integration, within a single miniaturizable platform, of molecular recognition and transduced signal generation, eliminating dependence on complex analytical pipelines and making point-of-care detection viable in primary-care environments [4]. Performance depends critically on the recognition element, the transducer, and, increasingly, the engineered surface in-between. Nano-carbon materials, including graphene and its oxides, carbon nanotubes, graphene and carbon quantum dots, nanodiamonds, and magnetic or hybrid composites, supply the biointerface in a growing share of cancer biosensors because they combine high specific area with surface chemistry rich in activated functional groups, which enables bioreceptor immobilization without additional activation steps, a direct advantage over conventional carbon platforms [5,6,7,8,9]; they often double as the transduction substrate in FET, electrochemical and optical readout architectures [10].

Convergence with the liquid-biopsy paradigm sharpens this relevance. Several nano-carbon platforms operationalize this convergence in liquid-biopsy and wearable formats, constituting the most direct interface between nano-carbon analytical capability and the clinical decision-making logic of liquid biopsy. Representative examples are discussed in Section 6 with full analytical context.

**Figure 1:**
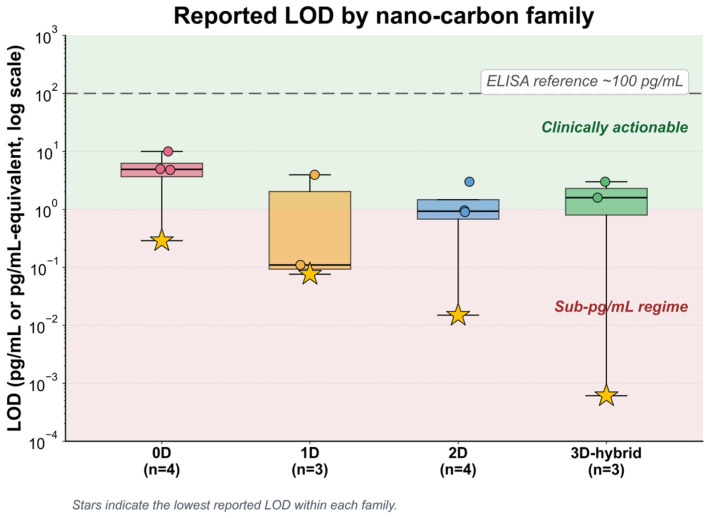
Reported limits of detection (LODs) by nano-carbon family among the 14 Section 3 studies that reported concentration-based LODs (0D, *n* = 4; 1D, *n* = 3; 2D, *n* = 4; 3D-hybrid, *n* = 3). Box plots are shown on a log-scaled LOD axis, and gold stars indicate the lowest reported LOD within each family. The dashed line denotes a nominal ELISA reference level (≈100 pg/mL), while the shaded pg/mL–ng/mL region represents the broader translational concentration band used in this figure.

**Figure 2:**
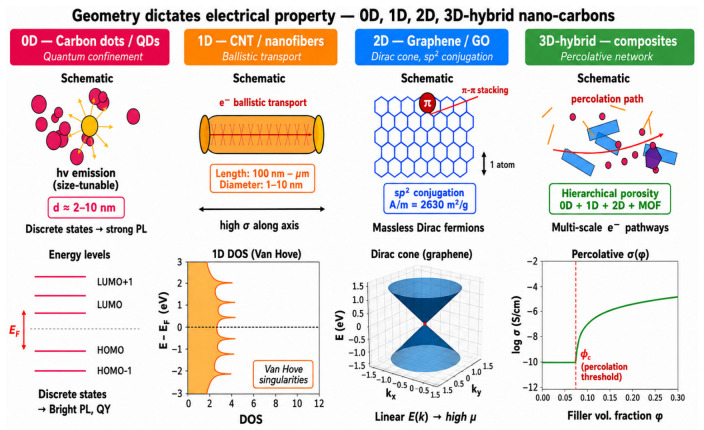
Reported LODs by functionalization route. The nano-carbon family is color-coded, and the marker shape encodes Evidence Level. Low LODs are observed for both covalent and non-covalent strategies, while Strong–Clinical evidence remains sparse across coupling chemistries.

**Figure 3:**
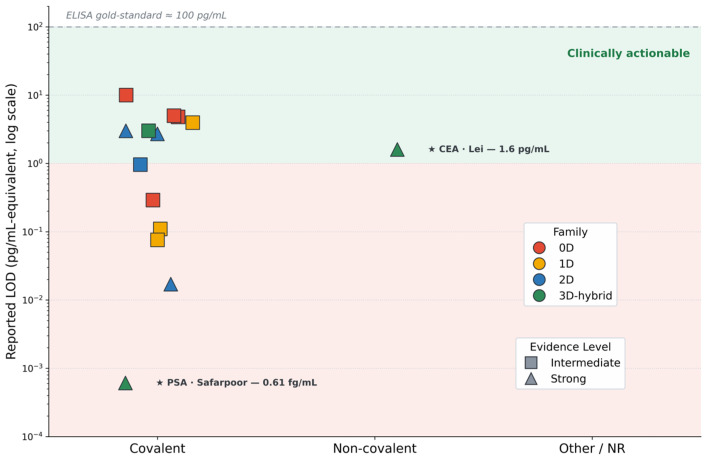
Covalent vs. non-covalent functionalization route × reported LOD: family is color-coded; marker shape encodes Evidence Level. The key take-home is that the lowest reported detection limits are distributed across both covalent and non-covalent functionalization routes rather than being confined to one, and that Strong–Clinical evidence level is sparse regardless of coupling chemistry, indicating that functionalization route selection does not by itself determine translational maturity.

The literature is asymmetric. Proof-of-concept reports dominate, frequently announcing femtomolar or attomolar LODs in buffer or spiked matrices, while validation against orthogonal clinical comparators, performance in undiluted human matrices, reproducibility across independent fabrications, and operational stability under field conditions are reported irregularly. Five bottlenecks, protein corona, batch-to-batch reproducibility, scalable synthesis, regulatory compatibility, and workflow integration, recur across families and modalities and are developed quantitatively. The distribution of reported LODs across nano-carbon families, illustrated in Figure 1, reveals that analytical sensitivity does not scale monotonically with platform dimensionality, a finding that motivates the matrix-based analysis developed. Figure 1, Figure 2 and Figure 3 share a consistent visual grammar: nano-carbon family is encoded by color, Evidence Level by marker shape, and the limit-of-detection axis uses a log scale; reading the three panels together gives a composite view of how material geometry, transport mechanism, and functionalization strategy jointly shape the analytical and translational landscape.

A scoping review is the appropriate design for this question, and three features of the current evidence support that choice. First, the nano-carbon biosensor literature for cancer detection published between 2024 and 2026 is not homogeneous enough in comparable intervention, standardized outcome, and defined target population to sustain a PICO question or a quantitative synthesis of effect sizes, which are the conditions a systematic review requires [11,12]. Second, the methodological variation across material families, recognition strategies, transduction principles, and biological matrices is itself what the review sets out to map, since a scoping approach is meant to chart such diversity rather than treat it as a source of bias to be controlled [13,14]. Third, the review seeks to locate conceptual and methodological gaps rather than to estimate effects or issue evidence-graded clinical recommendations, which is the purpose for which the scoping format was developed [12,15].

The objective of this review is to map how nano-carbon biointerfaces are being designed and applied as biosensors for cancer in the 2024–2026 biennium, and to identify the material, functional and translational determinants of the transition from proof-of-concept to clinical-grade evidence. The manuscript moves from protocol and analytical map (Section 2, Section 3, Section 4, Section 5 and Section 6) through translational diagnosis and bottleneck quantification (Section 7 and Section 8) to strategic perspectives and a proposed reporting checklist (Section 9 and Supplementary Material S2).

### 1.1. Background and Rationale

The upstream shift in detection, from symptomatic to asymptomatic windows, is the single intervention with the greatest demonstrated impact on outcome [3], yet its systematic delivery at scale in primary-care settings remains constrained by the analytical and logistical requirements of existing reference-standard methods. Nano-carbon biointerfaces have emerged as a structurally versatile platform for cancer-biomarker detection; π-stacking, edge-defect chemistry and quantum confinement translate molecular binding events into measurable signals through mechanisms that conventional carbon substrates cannot replicate, enabling miniaturized, integrated sensing without dependence on complex analytical pipelines. This asymmetry, analytical maturity without translational evidence, is precisely what motivates a structured mapping exercise of methodological and translational status, and what the Translational Readiness Matrix (Section 2.2) is designed to expose.

### 1.2. Why a Scoping Review

This review maps the current state of the field, identifying where progress has consolidated and where gaps remain unresolved rather than estimating a pooled effect size. This aim corresponds to the scoping review format and differs from the quantitative synthesis of a meta-analysis [15,16].

The included studies are too heterogeneous for meta-analytic pooling. They differ substantially in nano-carbon family, surface chemistry, biorecognition strategy, transduction modality, biological matrix, and reported outcome metrics. This heterogeneity is structural rather than incidental, since the field has not yet established a single platform architecture, biomarker class, or validation standard. In this setting, a pooled effect-size estimate would carry little interpretive value, because the variance between studies reflects genuine diversity in design rather than measurement error that averaging could remove. A scoping review suits these conditions, since its purpose is to map the breadth of evidence, identify gaps, and orient subsequent systematic reviews and primary research, consistent with JBI 2024 and PRISMA-ScR 2018 [12,15].

The analytical synthesis is operationalized through the Translational Readiness Matrix (Section 2.2 and Section 7), which serves as the structured instrument for categorizing advances, where the field has demonstrated consistent analytical performance, and gaps, where translational evidence is absent, incomplete, or unreported. This instrument transforms the descriptive map into an analytical product: not a catalog of what exists, but a diagnosis of what the field has achieved and what it still needs to demonstrate.

We adopt the current JBI scoping-review framework, the PRISMA-ScR 2018 reporting checklist, and the Population–Concept–Context (PCC) eligibility scheme, declaring a priori that no critical appraisal of individual sources is performed, a methodological decision fully justified in Section 2.2.

The restriction of the included studies to the 2024–2026 biennium reflects a deliberate scope decision, not an incidental search boundary. The nano-carbon biosensor field for cancer has foundational studies dating back more than a decade; those earlier works are excluded from the primary set of studies for two methodological reasons. First, they belong to a phase of the field characterized by a qualitatively different degree of technical and reporting heterogeneity, which makes longitudinal comparison of translational maturity across periods analytically unreliable. Second, the purpose of this review is to diagnose the current state of the field and to identify the bottlenecks that prevent progression to clinical practice, an objective for which the 2024–2026 window is the most analytically informative. The resulting interpretive limitation is explicit; the bottlenecks identified here may or may not represent improvements relative to earlier periods, a comparison that falls outside the scope of this review but constitutes a priority agenda for future longitudinal synthesis.

### 1.3. Operational Definitions

For consistency across charted studies, we define the following operational terms. Nano-carbon biointerface: the assembled architecture in which a carbon nanomaterial from one of the four dimensionality classes (Table 1) contacts a biorecognition element and a transduction substrate to convert a cancer-biomarker binding event into a measurable signal. Translational maturity: the position of a charted study within a five-stage trajectory (Proof of Concept; Analytical Validation; Real-matrix Validation; Preclinical; Clinical) intersected with Evidence Level (Low, Intermediate, or Strong), operationalized as the Translational Readiness Matrix in Section 2.2. Evidence Level is operationalized as quantitative thresholds (at least 15–20 independent replicates, recovery within 80–120%, and a linear R^2^ of at least 0.99), applied uniformly across studies regardless of biomarker, family, or transduction. Technological sovereignty: the capacity of a country or jurisdiction to develop, manufacture, certify, and deploy a technology with sufficient independence from external choke-points; the term is used throughout this review in a systems-capacity sense, not in any geopolitical or normative sense. Sovereignty domain: the cluster of supply-chain, regulatory, manufacturing, and deployment dimensions that characterizes the locus where translational gaps concentrate (Section 8).

### 1.4. Review Objectives and Research Questions

Three research questions structure the review. RQ1 asks which nano-carbon families, surface chemistries, and transduction modalities define the current design space. RQ2 asks how analytical performance varies across biological matrices and what that variation reveals about modality choice. RQ3 asks where the included studies sit on the translational trajectory and which bottlenecks most systematically constrain the transition from proof of concept to clinical readiness. These questions are addressed thematically across Section 3, Section 4, Section 5, Section 6 and Section 7 and integrated in Section 8 and Section 9.

## 2. Materials and Methods

This scoping review follows the JBI methodology for scoping reviews [12,14], with reporting per the PRISMA-ScR checklist [16] (a completed item-by-item PRISMA-ScR conformance map is provided in Supplementary Material S1) and PRISMA 2020 flow diagram conventions [17]. The a priori protocol, including PCC eligibility criteria, search strategy across six databases (PubMed/MEDLINE, Scopus, Web of Science, IEEE Xplore, ScienceDirect, and LILACS/DeCS BVS), three-stage screening rubric (Rayyan-mediated) [18], and per-section extraction templates, is reported in Supplementary Material S2. The PRISMA-ScR flow from 1689 initial records to 191 charted studies, with exclusion reasons by stage, is in Supplementary Material S2.8, Figure S1.

### 2.1. Population, Concept, Context (PCC) Framework

The review scope was defined using the PCC framework [12]:

Population (P): Adults and adolescents with confirmed or suspected solid tumor or hematological cancer, for whom a biomarker measurement is reported. Preclinical animal studies were excluded from the primary set of studies but retained as contextual references when they informed biomarkers or matrix selection.

Concept (C): Biosensor architectures incorporating one or more nano-carbon families, carbon dots, graphene/GO/rGO, carbon nanotubes, fullerenes, nanodiamonds, MXene-carbon hybrids or 3D composites, coupled to a biorecognition element (antibody, aptamer, peptide, nucleic acid probe or MIP) for cancer biomarker detection. Nano-carbon must function as an active component of the sensing layer, not merely as an inert substrate.

Context (C): Clinical, point-of-care, wearable, or liquid-biopsy applications targeting tumor biomarkers, proteins, microRNAs, circulating tumor cells/DNA, exosomes, and volatile organic compounds, in publications from 2024 to 2026.

No critical appraisal of individual sources was performed, consistent with JBI scoping-review methodology [12] and PRISMA-ScR item 12 [16], which direct scoping reviews toward evidence mapping rather than effect-size synthesis. The Translational Readiness Matrix (Section 2.2) was applied in its place as a structured descriptive instrument.

### 2.2. Translational Readiness Matrix

The Translational Readiness Matrix was developed to serve a function that standard critical-appraisal instruments cannot adequately perform in these studies. Tools designed for diagnostic accuracy studies, QUADAS-2, STARD, MINIMAR, require a defined clinical outcome and a validated reference comparator; fewer than 10% of the 191 charted studies satisfy those conditions. Applying them to the full set of studies would classify approximately 90% of studies as high risk of bias, a technically correct but analytically uninformative result for a field in which preclinical development dominates. The Matrix addresses this by stratifying studies according to translational stage rather than study quality in the conventional sense.

The Matrix is defined by two axes. The horizontal axis captures Evidence Level, Low, Intermediate or Strong, according to explicit per-cell criteria derived from established normative standards: Low (fewer than five independent replicates or no inter-batch variability quantified); Intermediate (five to fourteen replicates, recovery reported, R^2^ reported, no fabrication-scale validation); Strong (fifteen or more independent replicates, recovery 80–120% demonstrated in an undiluted or real clinical matrix, R^2^ ≥ 0.99, inter-batch CV ≤ 10% across ≥5 independent fabrications). The numerical thresholds are not arbitrary: the *n* ≥ 15–20 replicate floor derives from IUPAC (2002) and ISO 5725-1 recommendations for method precision studies; the 80–120% recovery window follows the FDA Bioanalytical Method Validation Guidance (2018) and the EMA Guideline on Bioanalytical Method Validation; the R^2^ ≥ 0.99 linearity criterion follows CLSI EP06 (Evaluation of the Linearity of Quantitative Measurement Procedures); and the CV ≤ 10% inter-batch threshold reflects the FDA reproducibility criterion for diagnostic submission. The three primary thresholds (replication, spike-recovery, and linearity) are cross-listed as operational definitions in Section 1.3 for reference throughout the review; the inter-batch CV ≤ 10% across ≥5 fabrications and the undiluted/Real-matrix recovery requirement are the refinements that distinguish the Strong Evidence Level from Intermediate.

Studies that do not meet the Strong Evidence Level criteria are not classified as low quality; they are classified as being at an earlier translational stage, which is the intended function of the instrument. The vertical axis captures the translational stage, adapted from the Technology Readiness Level scale: Proof of Concept; Analytical Validation; Real-matrix Validation; Preclinical; Clinical. Each study was positioned in the resulting 3 × 5 matrix based on its strongest reported endpoint. Classification was performed by two independent raters; boundary cases were resolved by consensus. Full cell-by-cell criteria, the rater-agreement protocol, the inter-rater agreement coefficient (Cohen’s kappa), and the consensus mechanism are detailed in Supplementary Material S2.6.

## 3. Nano-Carbons as a Technological Frontier

Platforms are grouped by dominant structural dimensionality (0D–3D) because dimensionality corresponds to differences in charge transport and electronic properties that determine the sensing performance of each family. In zero-dimensional (0D) platforms, electronic transport is governed by quantum confinement, energy states are discrete, optical transitions are size-dependent, and reduced electron–phonon coupling favors high photoluminescence efficiency, which underpins optical and fluorometric transduction. In one-dimensional (1D) platforms, single-walled carbon nanotubes conduct electrons in the ballistic regime along the tube axis when the device length falls below the mean free path (~1 µm for SWCNTs at room temperature), yielding quantized conductance with negligible resistive dissipation, a property that enables ultrasensitive FET biosensors.

In two-dimensional (2D) platforms, the hexagonal sp^2^ network of graphene produces a band structure with a linear crossing at the K and K′ points of the Brillouin zone (the Dirac cone), where charge carriers behave as massless Dirac fermions; the electric field of a single adsorbed biomolecule modulates carrier density detectably. In three-dimensional hybrid (3D) architectures, the combination of nano-carbon phases with secondary components (metal oxides, MXenes, MOFs) creates multi-scale percolative networks in which conductive pathways emerge through the dispersed phase, transport arises at the percolation threshold, and the sensor’s linearity window is defined. Figure 2 maps these geometry-dictated electrical signatures across the four families.

**Figure 4:**
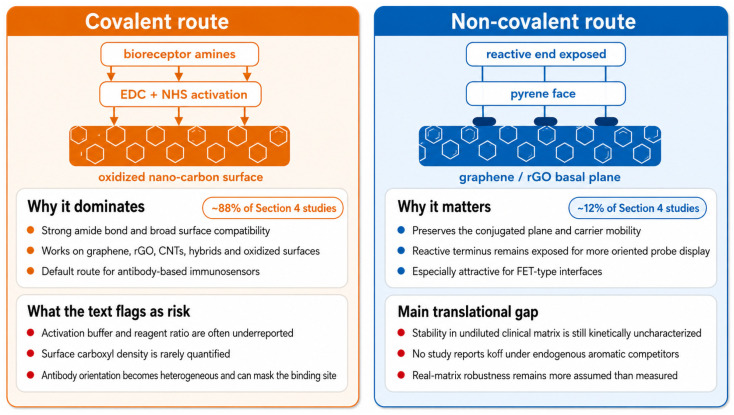
Schematic comparison of covalent EDC/NHS coupling and non-covalent π–π/pyrene-mediated biofunctionalization on nano-carbon surfaces. Key trade-offs are summarized below each panel; study-level route classifications are provided in Supplementary Material S3, Section 4 charting.

After title and abstract screening, 26 records were assessed for this section, of which 19 met full-text eligibility criteria (Supplementary Material S2.8, Figure S1). Seven studies were excluded because nano-carbon acted only as a payload carrier rather than as the sensing interface, or because the biomarker was not disease-specific. Retained studies are summarized in Supplementary Material S3, Section 3 charting, and discussed below by material architecture. Cross-family trends are synthesized in Section 3.5 and Figure 4; LOD distribution across families is illustrated in Figure 1.

### 3.1. Zero-Dimensional Carbons

Four studies used zero-dimensional carbon nanomaterials, carbon dots (CDs), carbon quantum dots (CQDs), graphene quantum dots (GQDs), and nanodiamonds as the central sensing component. Quantum confinement in these materials emerges when at least one spatial dimension approaches the Bohr radius of the exciton (~1–10 nm for carbon-based quantum dots). Below this threshold, the energy continuum of the conduction and valence bands breaks into discrete quantized levels, and the optical gap scales inversely with the square of particle diameter (E ∝ 1/d^2^). In practice, this means (i) the photoluminescence emission peak can be tuned by synthesis; smaller GQDs emit in the blue-UV, larger ones in the green-yellow, enabling multiplexed sensors with distinct spectral windows without receptor substitution; (ii) the fluorescence quantum yield of CDs and N-CDs can reach 40–80%, exceeding most organic fluorophores, and is sensitive to analyte adsorption at the surface via quenching or enhancement; and (iii) surface functional groups (–COOH, –OH, –NH_2_) modulate the surface states that contribute to emission, making the spectral response sensitive to pH, solvent polarity and target biomolecule binding.

These three attributes position 0D materials as predominantly optical and fluorometric platforms within these studies. The size-tunability of the emission peak is a direct translational advantage; a single synthesis batch adjusted to a target diameter can be spectrally differentiated from a second batch targeting a different biomarker, enabling multiplexed detection without receptor substitution, a design logic that antibody-based platforms cannot achieve without independent labeling chemistries. The quantum yield advantage of N-doped CDs over conventional organic fluorophores (40–80% versus typically 10–40% for rhodamine derivatives) translates into higher signal-to-noise ratios at equivalent analyte concentrations, which is particularly relevant for low-abundance cancer biomarkers. Surface-state sensitivity to pH and bound biomolecules introduces a matrix-dependent spectral response that must be characterized and controlled in complex biological matrices such as undiluted serum or plasma. The four studies operationalized this potential across distinct interface designs.

Anbalagan et al. [19] used cow-urine-derived CDs in an enzyme-labeled antibody system for carcinoembryonic antigen (CEA) detection (LOD 10 pg/mL; linear range 0.5–50 ng/mL). Amir et al. [20] applied nitrogen-doped CQDs on graphite for HER2 detection (LOD 4.8 pg/mL; linear range 0.1–1 ng/mL). Olorundare et al. [21] combined nanodiamonds with gold nanoparticles for HER2 detection (LOD 0.29 pg/mL; linear range 1 pg/mL–50 ng/mL). Cotchim et al. [22] developed a hybrid interface comprising GQDs and plant-derived carbon for PSA detection (LODs of 0.005 ng/mL [5 pg/mL]; linear ranges of 0.0125–1 ng/mL and 1–80 ng/mL). All four platforms achieved low-picogram-per-milliliter detection limits; in Supplementary Material S3, these four 0D studies are classified as Intermediate Evidence Level rather than Low Evidence Level.

### 3.2. One-Dimensional Carbons

Five studies used carbon nanotubes as the main conductive biointerface, predominantly oxidized or carboxylated multi-walled carbon nanotubes [22,23,24,25,26]. The exceptional electrochemical performance of CNT-based biosensors derives from the ballistic transport regime that characterizes metallic SWCNTs and, to a lesser extent, low-defect-density MWCNTs. In ballistic transport, electrons traverse the conductive channel without appreciable inelastic scattering; channel conductance is quantized as G = 2e^2^/h × N, where N is the number of available conduction channels. For a metallic (6, 6) SWCNT at room temperature, N = 2, yielding a minimum conductance of ~155 µS, a value intrinsic to the nanotube structure and independent of length, provided the device length remains below the mean free path. In MWCNTs, inner shells contribute additional channels, but the defect density introduced by functionalization (–COOH, –OH) partially reduces ballistic character. The net consequence for biosensing is twofold: exchange current density at the electrode–solution interface exceeds that of glassy carbon or conventional graphite by orders of magnitude, which underpins the femtomolar–attomolar LODs reported by 1D electrochemical platforms; and the heterogeneous electron-transfer rate constant (k^0^) responds to molecules adsorbed along the nanotube axis, enabling label-free detection.

In these five systems, the nanotube network provided both an electron-transfer pathway and a chemically functional scaffold for biomolecule immobilization. Additional components, layered hydroxides, metal-organic frameworks, metal hydroxides, quantum dots, noble-metal nanoparticles, or MXene were incorporated to improve signal amplification, selectivity, or surface reactivity. Detection limits were generally in the picogram-per-milliliter range. Among these, Shamsazar et al. [25] reported the lowest detection limit, with an LOD of 0.076 pg/mL for CEA (linear range 0.0001–2 ng/mL).

### 3.3. Two-Dimensional Carbons

Five studies explored two-dimensional carbon-based interfaces, predominantly graphene-derived materials. The functional properties of these platforms derive from the hexagonal sp^2^ carbon network, in which each atom contributes one delocalized π electron to a conjugated system extending across the entire two-dimensional sheet. This conjugation produces two sensorially relevant effects: (i) the graphene surface is electronically sensitive to local electrostatic perturbations; a single elementary charge adsorbed on a high-quality graphene FET is detectable as a conductance change, which underpins the sub-femtomolar detection limits reported for 2D FET platforms; and (ii) the delocalized π electron density of the basal plane provides non-covalent adsorption sites via π–π stacking for aromatic molecules, including nucleic acid bases and pyrene-based linkers, enabling non-covalent biofunctionalization without disrupting the band structure. In GO and rGO, the insertion of oxygenated functional groups (epoxy, hydroxyl, carboxyl) partially interrupts sp^2^ conjugation, opening an energy gap that reduces conductivity but increases surface reactivity for covalent biomolecule immobilization.

This group was the most analytically diverse, including one theoretical study on doped carbon nanoribbons for volatile gastric cancer biomarkers [27] and four experimental platforms based on graphene, rGO, or laser-induced graphene targeting neuron-specific enolase, autocrine motility factor, microRNA panels, and alpha-fetoprotein or estradiol [28,29,30,31]. The experimental platforms generally occupied the Intermediate-to-Strong Real-matrix evidence cells of the Translational Readiness Matrix, whereas the theoretical study remained at the pre-analytical stage. The relationship between functionalization route, reported detection limit, and evidence level is illustrated in Figure 3.

### 3.4. Three-Dimensional and Magnetic–Carbon Hybrids

Five studies used three-dimensional or hybrid nano-carbon architectures combining carbon materials with magnetic phases, noble-metal components, transition-metal quantum dots, or a second carbon family [32,33,34,35,36]. These architectures build electronic pathways across multiple scales through two complementary mechanisms. At the nanometric level, the interface between the nano-carbon and the secondary phase (MXene, Fe_3_O_4_, MOF, gold nanoparticles) creates heterojunctions with band alignment favorable to charge transfer; when the Fermi potentials of the two phases differ, spontaneous charge redistribution at the interface generates an internal electric field that accelerates redox electron transfer, the mechanism underlying the superior exchange current densities consistently reported for 3D hybrids relative to single-component platforms. At the mesoscopic level, dispersion of the secondary phase within the nano-carbon matrix modifies the geometry of conductive pathways; nanotubes or graphene sheets that individually form disconnected conductive segments become electrically bridged by the secondary nanocomposite, increasing the density of continuous conductive paths throughout the biointerface.

This bridging effect is governed by percolation. In a random network of conductive particles dispersed in an insulating matrix, a critical volumetric fraction of conductive phase, the percolation threshold (φ_c_), must be exceeded before at least one continuous conductive pathway spans the full volume. For spherical particles φ_c_ ≈ 16–20%; for high-aspect-ratio nanotubes, φ_c_ can fall below 0.1%, explaining why minimal CNT additions to metal-oxide composites produce dramatic conductivity gains. In biosensor design, operating above φ_c_ distributes current uniformly across the biointerface, reduces charge-transfer resistance (R_CT_), and expands the electroactive area. The LOD heterogeneity observed within the 3D family in these studies (Table 1) is partially attributable to unreported variation in nano-carbon volumetric fraction across studies, a variable that rarely appears in the characterization sections of primary articles.

The five platforms in this group included: a nanotube-bridged MXene network for CEA detection [32]; a magnetic rGO immunosensor for HER2-positive cells [33]; a magnetic nanotube/graphene system for cancer antigen 15–3 in serum [34]; a graphene oxide/tungsten disulfide hybrid for gastric cancer biomarkers [35]; and MXene quantum dots for PSA [36]. Several were positioned in the Strong Real-matrix evidence cell, particularly those incorporating serum testing; others remained at Intermediate Analytical Validation.

### 3.5. Cross-Family Synthesis

Before presenting the cross-family patterns, a structural limitation must be stated explicitly. The 19 platforms in this section, and the 191 studies in the full set of studies, do not constitute a homogeneous set that can be directly compared on any single performance axis. They differ in target analyte (proteins, nucleic acids, exosomes, CTCs, VOCs), whose physiological concentrations span more than ten orders of magnitude; in biological matrix (buffer, spiked serum, undiluted plasma, saliva, urine); in signal readout (electrochemical, optical, FET); and in fabrication complexity. Direct LOD comparisons across these dimensions have limited interpretive value without matrix and analyte normalization, a constraint that the Translational Readiness Matrix addresses by stratifying studies along axes independent of the specific analyte–matrix combination.

With that caveat stated, three patterns emerge from the included studies. First, analytical sensitivity is not a function of dimensionality. The lowest reported LODs appear in both 0D and 3D-hybrid platforms [21,36], confirming that surface chemistry, recognition strategy, and signal amplification matter at least as much as material geometry. Second, each family aligns with particular transduction strategies: 0D materials predominantly support optical and fluorometric readouts; 1D and 3D-hybrid systems favor electrochemical architectures; 2D graphene-derived platforms span the broadest range, from FET arrays to electrochemical and theoretical screening formats [27,28,29,30,31].

Third, translational maturity is uneven; Real-matrix evidence is concentrated among 2D experimental systems and 3D hybrids, while several 0D platforms remain at the proof-of-concept stage. To clarify how functionalization chemistry shapes the performance of nano-carbon biointerfaces. Table 1 compares covalent EDC/NHS coupling with pyrene-mediated π–π stacking across five mechanistic criteria: sp^2^ network integrity, bond stability, bioreceptor orientation, inter-batch reproducibility, and electron-transfer kinetics.

## 4. Surface Chemistry and Biofunctionalization

This section maps the surface chemistry and biorecognition strategies identified across the 31 primary studies retained in the Section 4 working set of studies, plus three outside-set of studies exemplars retained for narrative reference; all 34 entries are tabulated in Supplementary Material S3, Section 4 charting (three exclusions at Step 3 for being review-only or addressing non-cancer biomarkers). The recurring observation is that surface chemistry is treated instrumentally; biorecognition performance is reported in detail, whereas coupling chemistry, its yield, hydrolysis kinetics, and protein-corona effects are reported only intermittently. The section is organized by coupling route (Section 4.1 and Section 4.2), followed by antifouling and recognition-element integration (Section 4.3), and closes with the biointerface chain as an integrative argument (Section 4.4).

### 4.1. Covalent Coupling: EDC/NHS

Carbodiimide-based coupling, using EDC with NHS or sulfo-NHS, was the most frequent immobilization strategy in the Section 4 charting set, appearing as the primary route in 16 of 34 studies (47%) and in 18 of 34 studies (53%) when mixed EDC/NHS routes are included. Its prevalence reflects its apparent simplicity; carboxyl groups on the nano-carbon surface are activated and linked to amine-containing biomolecules, forming covalent amide bonds with a dissociation energy of ~350 kJ/mol that are stable across pH 4–9 and temperatures of 4–40 °C.

However, this apparent standardization conceals important sources of variability. Activation buffer conditions, composition, pH, ionic strength, reagent ratio, and reaction time were reported incompletely in most studies. The density of carboxyl groups on the carbon surface was rarely quantified, despite varying substantially across suppliers, oxidation methods, and post-treatments. Two studies using commercial rGO from different suppliers reported coupling yields differing by approximately one order of magnitude, a direct consequence of uncharacterized surface heterogeneity. A further limitation is orientation control; EDC/NHS reacts with lysine ε-amines distributed across the antibody surface, producing mixed orientations that risk occluding the antigen-binding site and reducing effective recognition capacity. Treating EDC/NHS as a default protocol without characterizing these variables obscures a major determinant of reproducibility. Figure 4 contrasts this covalent coupling route with non-covalent π–π stacking, highlighting how different immobilization strategies shape the stability and organization of the biointerface.

### 4.2. Non-Covalent: π–π and Friends

Non-covalent functionalization represents the second most frequent immobilization route identified in the Section 4 dataset. Among these strategies, π–π stacking mediated by pyrene-based linkers is the archetypal approach for sp^2^ carbon interfaces. The most common linker is 1-pyrenebutanoic acid succinimidyl ester, reported as PBSE, PyBSE, PBASE, or PASE depending on nomenclature.

The main advantage of this route is that it enables bioreceptor immobilization without forming a covalent bond directly with the graphene or graphitic basal plane. As a result, the sp^2^-conjugated network is better preserved than in oxidation-dependent covalent approaches, helping maintain the electronic properties critical to field-effect transistor platforms. This distinction is especially relevant for FET biosensors, in which basal-plane disruption, oxidation heterogeneity, or defect generation may introduce charge-scattering sites and reduce carrier mobility, transconductance, and signal reproducibility. In pyrene-mediated functionalization, the aromatic pyrene moiety adsorbs onto the sp^2^ carbon surface through π–π interactions, whereas the succinimidyl ester terminus remains available for subsequent reaction with primary amines on proteins, antibodies, enzymes, or other bioreceptors. This architecture separates the carbon–linker interaction from the bioreceptor-coupling chemistry and avoids direct activation of heterogeneous surface carboxyl groups. However, because NHS chemistry still reacts with accessible amines on the bioreceptor, the resulting immobilization should be described as more surface-preserving rather than fully orientation-controlled.

The principal translational limitation of this route is not initial immobilization efficiency but kinetic stability under clinically realistic conditions. Although pyrene–carbon interactions are sufficiently strong for many buffer-based assays and have been widely used in graphene and carbon nanotube biosensors, the dissociation kinetics of pyrene linkers in undiluted biological matrices remain insufficiently characterized. In particular, the effects of serum proteins, lipids, and endogenous aromatic competitors on linker retention, receptor density, and long-term signal stability were not systematically evaluated in the Section 4 studies. Therefore, π–π stacking offers a clear advantage in preserving electronic performance, but its translational robustness depends on matrix-specific stability testing. Table 2 contrasts covalent EDC/NHS and non-covalent pyrene-mediated π–π stacking across five criteria directly relevant to translational biosensing.

Taken together, these five criteria indicate that neither route dominates unconditionally: EDC/NHS offers superior bond stability and is compatible with the widest range of nano-carbon surfaces but introduces reproducibility variables that are rarely controlled; π–π stacking preserves transduction performance and enables oriented immobilization, but its kinetic stability in undiluted clinical matrices remains the principal translational limitation of this route.

The fundamental variable that remains systematically uncharacterized is the dissociation rate constant (k_off_) of the pyrene–carbon complex under real biological matrix conditions. In aqueous buffer, k_off_ for π–π stacking is reported to be in the range 10^−3^–10^−2^ s^−1^, slow enough to keep the functional layer stable over a 30–60 min assay. In complex biological matrices, however, endogenous aromatic competitors such as bilirubin, phenylalanine, and circulating nucleotides can displace pyrene from the basal plane in a concentration-dependent, nonlinear manner, thereby unpredictably accelerating k_off_. No study in Section 4 reports k_off_ in the presence of endogenous aromatic competitors, meaning that the true stability of the π–π biointerface in the undiluted clinical matrix remains undetermined for this route.

**Figure 5:**
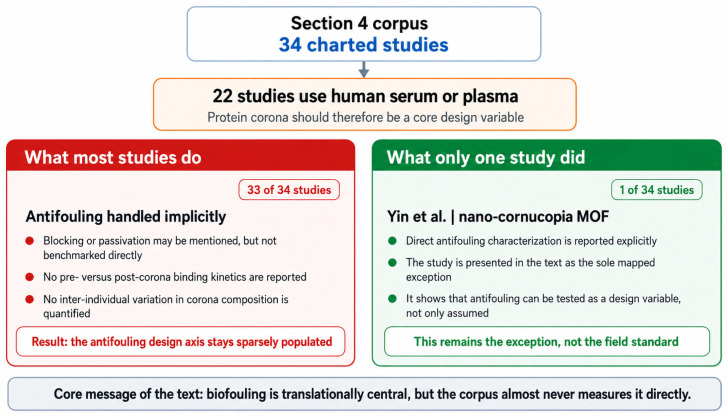
Antifouling architectures identified across nano-carbon biosensor platforms exposed to human serum or plasma matrices. Representative strategies to mitigate protein-corona formation and nonspecific adsorption are summarized, highlighting the limited direct characterization of antifouling performance across the charted studies.

Less frequent non-covalent architectures include biotin–streptavidin pairs for probe-exchangeable formats, electrostatic adsorption on charged carbons for rapid protein screening, and hydrazone-mediated orientation via oxidized antibody glycans. Layer-by-layer assemblies, MOF/LDH intermediate scaffolds, and nano-cornucopia MOF architectures extend this group by using nano-carbon as one component in a stratified architecture rather than as a flat receptor surface, thereby trading spatial control over probe density and orientation for additional assembly steps. Figure 5 illustrates the architectural contrast between these two routes and reports a set of study frequencies for each.

### 4.3. Antifouling, Recognition Elements, and Matrix Performance

Twenty-two of the 34 charted papers used human serum or plasma, either spiked or clinical, as the analytical matrix. The protein corona is therefore the dominant matrix effect in this section and forms the core of Bottleneck 1, developed in Section 7.1. Despite this centrality, only one study, the Yin nano-cornucopia MOF platform [34], directly reported antifouling characterization; the remaining studies addressed protein adsorption only implicitly, if at all. Neither pre- versus post-corona binding kinetics nor inter-individual variability in corona composition was quantified in the Section 4 set, and the same gap extends to 43 of 87 studies in the broader Section 7 pool.

The choice of recognition element was closely linked to the coupling route and antifouling requirements. Antibodies remained the most common recognition element, compatible with carbodiimide coupling, hydrazone chemistry, and Protein A/G-mediated orientation, but their performance is sensitive to surface orientation, coupling density, and matrix-induced fouling, exactly the variables most frequently underreported. Aptamers were selected when greater surface organization or matrix tolerance was needed; their smaller size and chemical programmability enable immobilization on passive or zwitterionic surfaces that reduce nonspecific adsorption by abundant serum proteins, an advantage directly relevant to undiluted plasma assays. Molecularly imprinted polymers have emerged as a recognition layer for targets less suited to antibody-based detection, small molecules, and selected nucleic acid-related targets, offering chemical robustness and synthetic tunability at the cost of requiring selectivity validation in complex matrices. The full per-study surface-chemistry audit is provided in Supplementary Material S3 (Section 4 charting).

Three limitations recurred across recognition strategies: incomplete description of carbodiimide activation conditions (pH, reagent ratio, biomolecule-to-surface stoichiometry); sparse antifouling validation despite frequent use of serum or plasma; and infrequent direct comparison with established clinical methods using the same samples, with statistical agreement rarely quantified. These limitations feed directly into the chain-of-decisions argument in Section 4.4 and the minimum reporting set proposed in Supplementary Material S2.1.

### 4.4. The Biointerface Chain

Biointerface design is not a sequence of independent choices. The selected nano-carbon architecture determines which coupling chemistries are accessible; the coupling route shapes the orientation, density, and accessibility of the recognition element, and these features, in turn, condition the transduction signal, matrix tolerance, and performance within a given biomarker class. Conversely, the intended clinical use case should guide the initial selection of material architecture, surface chemistry, and recognition element, a logic that the included studies apply inconsistently. Because serum and plasma matrices expose this chain to protein-corona formation and nonspecific adsorption, Figure 5 summarizes the antifouling architectures identified across the studies charted, while the broader reporting implications of limited antifouling characterization are considered in Section 7.2.

**Figure 6:**
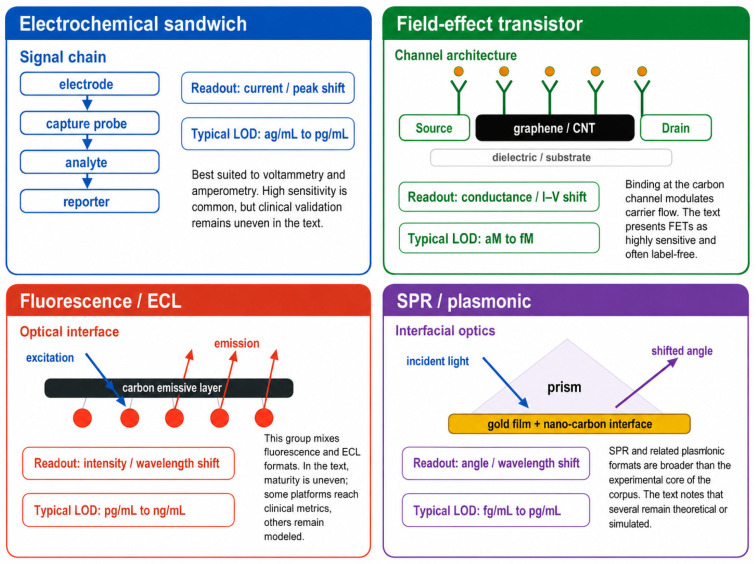
Main transduction principles used in nano-carbon cancer biosensors: electrochemical sandwich assays, field-effect transistor sensing, fluorescence/electrochemiluminescence readouts, and SPR/plasmonic detection. Panels summarize the sensing stack, readout mode, and typical LOD regime. Per-study LOD and linear-range data are provided in Supplementary Material S3, Section 5.

Liu et al. [37] illustrate both the coherence and the limits of this chain in a wearable, non-covalent format: laser-induced graphene patterned on flexible polyimide, combined with a molecularly imprinted polymer for target recognition in sweat, without antibodies, covalent coupling, or enzymatic labels. The design is synthetically robust, cold-chain-independent, and compatible with decentralized testing, but the same architecture raises questions about selectivity drift, surface fouling, and polymer swelling under prolonged use. The translational value of the platform, therefore, depends not only on its LOD but also on the coherence of the entire biointerface chain under the conditions of intended use. This chain ultimately converges on the transduction strategy, which determines how recognition at the nano-carbon biointerface is converted into a measurable analytical signal. Figure 6 summarizes the four dominant transduction principles identified across the included studies.

## 5. Transduction and Device Architecture

This section examines how nano-carbon biointerfaces were translated into measurable signals and device formats. Eighteen primary studies were retained after full-text screening (Supplementary Material S2.8, Figure S1); analytical details are provided in Supplementary Material S3. The studies are organized by transduction strategy (electrochemical, field-effect transistor, optical and photoluminescent, and hybrid/multimodal/wearable), with a critical synthesis in Section 5.5 that interprets each modality in relation to target analyte, biological matrix, and translational readiness.

### 5.1. Electrochemical Transduction

Electrochemical transduction exploits the high heterogeneous electron-transfer rate constants (k^0^) characteristic of CNT and graphene surfaces, the same ballistic and Dirac-cone transport properties established in Section 3, to convert redox events at the biointerface into measurable current or impedance signals. It was the most frequent transduction strategy in this section, represented by seven studies.

Most platforms used voltametric detection (DPV or SWV), reflecting the compatibility between nano-carbon materials and faradaic electron-transfer sensing. Carbon nanotubes, graphene derivatives, MXenes, and carbon dots that were combined with catalytic, metallic, or amplification components to improve signal intensity [38] reported a ratiometric internal-reference immunosensor for urinary NMP22 in bladder cancer, tested in real urine (LOD 3.33 fg/mL; linear range 0.01 pg/mL to 1000 ng/mL). Huang et al. [39] detected miRNA-21 on a graphene/graphene-oxide platform (LOD 3.18 fM; linear range 10 fM to 1 nM in buffer). Long et al. [40] targeted circulating tumor DNA with a carbon-dot nanozyme cascade (LOD 1.26 aM; linear range 5 aM to 50 nM). Yan et al. [41] developed a multiplexed array for simultaneous detection of CRP, CA-125, and CEA in real plasma (LODs of 0.005, 0.0198, and 0.007 ng/mL or U/mL, respectively; CRP linear range of 0.05 to 50,000 ng/mL). Zhang et al. [42] reported CEA detection using an MXene/MWCNT composite (LOD 0.015 ng/mL; linear range 0.050 to 200 ng/mL).

Two additional electrochemical formats were less frequent but analytically distinct. Nagdeve et al. [40] used an impedimetric graphene platform for miRNA-31 detection (LOD 70 pg/mL in buffer, ≈10^−11^ M; linear range 10^−11^ to 10^−6^ M), a label-free format that captures binding kinetics rather than redox current. Dang et al. [43] applied an amperometric rGO/Prussian-blue/platinum system for mesenchymal circulating tumor cells in spiked whole blood (LOD 0.1 cells/mL; linear range 1 to 1 × 10^5^ cells/mL).

Overall, these platforms reached very low detection limits across analyte classes. However, analytical sensitivity was not consistently accompanied by translational validation; only three studies reported testing in real clinical matrices [38,41,43], and inter-batch reproducibility was quantified in only four of the seven studies. These gaps are developed in Section 7.2.

### 5.2. Field-Effect Transistor Architectures

Field-effect transistor (FET) architectures transduce biorecognition directly as a change in channel conductance, without a redox label [44]; target binding at the gate shifts the surface potential and modulates the current through the semiconducting nano-carbon channel. Operation in the sub-threshold regime gives these devices their characteristic sensitivity, but it also makes them acutely dependent on Debye screening, so performance is governed by ionic strength and by the placement of the probe relative to the channel. Four studies in this section used FET transduction.

Liu et al. [45] built a probe-screened single-walled carbon nanotube FET for the breast cancer gene BRCA1, achieving 1.38 aM in human serum and whole blood (linear range: 10 aM to 100 fM; *n* = 16 real samples). Ali et al. [46] reported a carbon nanotube FET immunosensor for interleukin-6 in artificial saliva (LOD: 1.15 pg/mL; linear range: 1.0 to 300 pg/mL). Zhu et al. [47] extended the format to a multiplexed FET that co-detected KRAS-G12D and AKT2 circulating tumor DNA mutations (LODs of 5.7 aM and 3.0 aM; linear range of 10 aM to 100 fM). Yadav et al. [48] proposed a graphene nano-engineered, plasma-tailored dielectric FET for breast tumor cell sensing, but the device was evaluated only by simulation and reports no experimental LOD, positioning it in the Low/Pre-analytical tier of the Translational Readiness Matrix.

### 5.3. Optical and Photoluminescent Transduction

Optical transduction in nano-carbon platforms exploits the size-dependent photoluminescence of 0D materials, governed by the quantum confinement mechanism established in Section 3, as well as plasmon-resonance effects at carbon–metal hybrid interfaces. Six studies used light-based transduction, spanning fluorescence, electrochemiluminescence, surface plasmon resonance, and terahertz sensing. These approaches differed substantially in translational maturity; fluorescence and electrochemiluminescence were represented by experimental biosensors tested in biological matrices, whereas SPR and terahertz platforms were exclusively theoretical or simulated, a distinction that must be kept explicit when interpreting the group’s aggregate performance.

Among fluorescence-based studies, Li et al. [49] developed a label-free aptasensor for salivary exosomes from oral squamous cell carcinoma, using dual-scale amplification and clinical matrix testing (LOD: 100 particles/mL; linear range: 2.5 × 10^2^ to 5 × 10^8^ particles/mL). Aravind et al. [50] used fluorescent carbon dots to detect flavin adenine dinucleotide in plasma (LOD of 2.3 µM; linear range reported graphically in the original study). In electrochemiluminescence, Li et al. [51] reported a carbon-dot-based biosensor for BRAF V600E detection in thyroid-cancer-derived exosomes (LOD 0.5 fM; linear range 1 fM to 100 nM), a background-free format that reaches femtomolar sensitivity but remains laboratory-bound due to co-reactant requirements.

The remaining three optical studies were exclusively theoretical. Othman et al. [52] proposed a terahertz metasurface combined with machine-learning signal interpretation; Karki et al. [53] and Rafighirani et al. [54] explored SPR and absorber-based designs for cancer-related detection. None reported experimental LOD values. Their inclusion reflects an emerging design space rather than demonstrated analytical performance, and they are accordingly positioned in the Low/Pre-analytical cell of the Translational Readiness Matrix.

### 5.4. Hybrid, Multimodal, and Wearable Architecture

Several studies combined more than one architectural strategy, a device-level approach intended to improve robustness, multiplexing, or signal discrimination rather than simply reduce LOD. These included internal-reference electrochemical designs, multiplex electrode arrays, hybrid nano-carbon structures, machine-learning-assisted signal interpretation, and closed-loop wearable formats [37,38,42,47,52].

Internal-reference systems reduce analytical variability by comparing two signals within the same device; the ratiometric design reported by [38] Zhou et al. exemplifies this approach, normalizing the target signal against an internal standard to compensate for matrix-induced drift. Multiplex arrays enable simultaneous measurement of multiple biomarkers [41], including co-detected three cancer-associated proteins in real plasma [55] and co-detected three cervical-cancer-associated biomarkers using a screen-printed electrode array functionalized with a graphene/gold nanoparticle/chitosan composite and supported by machine-learning analysis. This design shows how multiplexed signals may improve diagnostic classification beyond single-biomarker thresholds, but it also introduces a translational challenge specific to algorithmic platforms; when interpretation depends on a multi-marker algorithm, validation must address algorithm stability, training-data representativeness, and post-deployment monitoring, not only analytical performance.

The single fully validated wearable platform in this section [37], a LIG/MIP sweat sensor for testosterone with microfluidic sample handling and closed-loop operation, illustrates both the translational promise of wearable nano-carbon architectures and the gap that separates them from laboratory-bound platforms; deformation tolerance, real-time operation, and on-body reproducibility are the dominant performance axes, and LOD against a clinical comparator is the validation criterion that most frequently goes unreported.

Hybridization is therefore not only a material feature but a device-level strategy. It does not, however, substitute for clinical validation; when multiple markers, algorithms or device components are assessed together, the validation burden increases proportionally.

### 5.5. Critical Synthesis: Where Architecture Meets Translation

**Figure 7:**
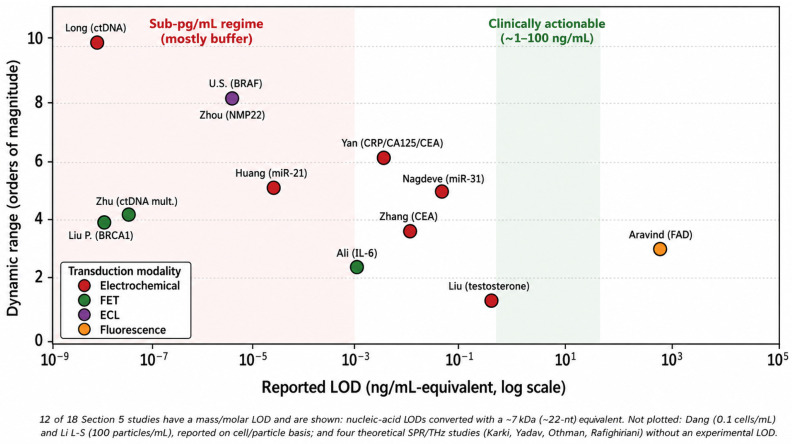
Relationship between reported LODs and linear range across Section 5 transduction modalities. The figure highlights the mismatch between ultralow analytical sensitivity and clinically relevant concentration windows. Green band = clinically actionable LOD window (≈1–100 ng/mL for proteomic markers; ≈pM for nucleic-acid panels). Red zone = sub-pg/mL regime, demonstrated in buffer only in these studies. Per-paper LOD values in Supplementary Material S3 (Section 5 charting).

Three patterns emerge from the eighteen studies in this section. First, LOD is the wrong primary metric for clinical translation when interpreted in isolation. The clinically actionable concentration window for proteomic cancer biomarkers is approximately 1–100 ng/mL, whereas nucleic-acid panels often operate in the picomolar range. As Figure 7 shows, the sub-pg/mL LODs most frequently reported in these studies fall largely below these clinically relevant windows and were demonstrated predominantly in buffer rather than in undiluted biological matrices. The operationally decisive parameters are LOQ, linear range, and matrix recovery. LOQ defines the lowest concentration that can be quantified with acceptable precision under real conditions; linear range defines the working window relevant to clinical concentrations; and recovery quantifies matrix-induced signal suppression or enhancement. Fewer than one-third of the eighteen studies in this section reported all three. This reporting gap is the most direct translational constraint identified in Section 5 and feeds directly into the minimum reporting set proposed in Supplementary Material S2.1.

**Figure 8:**
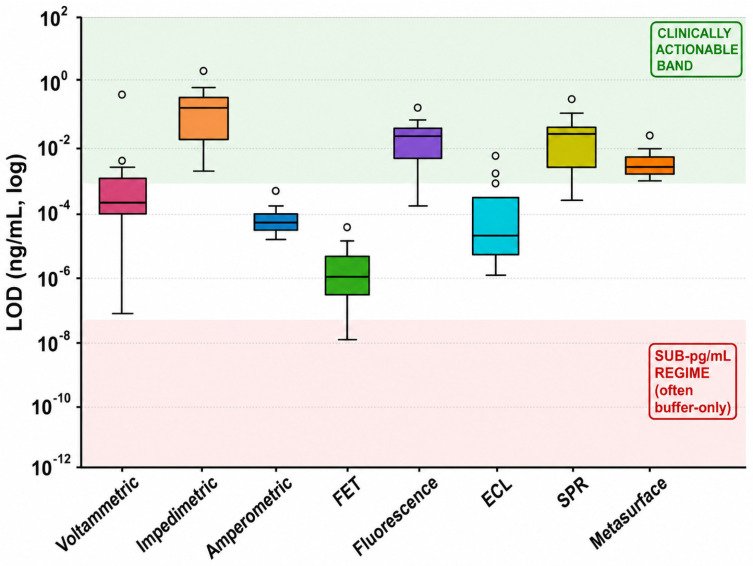
Reported LOD versus clinical Evidence Level across Section 5 studies. The empirical trend indicates that lower LODs were not consistently associated with stronger clinical evidence. The dashed gray line shows the hypothetical trend expected if analytical sensitivity were directly predictive of translational maturity.

Second, sub-femtomolar LODs are no longer the differentiating variable. Several studies reached femtomolar or sub-femtomolar sensitivity across voltametric, FET, and electrochemiluminescent modalities [40,45,51]. These analytical gains were not consistently matched by stronger clinical validation, and the platforms with the lowest detection limits were not necessarily those with the most advanced evidence. Figure 8 illustrates this directly; the empirical relationship between LOD and clinical evidence score is markedly flatter than would be expected if sensitivity alone predicted translational readiness.

Third, theoretical and simulated studies are concentrated in optical modalities. All three SPR/THz studies were exclusively computational, whereas the experimental evidence base is concentrated in electrochemical, FET, and wearable formats. This asymmetry is not a quality judgment; theoretical platforms can define design targets that experimental work subsequently validates. However, it must be explicitly stated when interpreting aggregate LOD distributions, since simulated LODs are not directly comparable to experimentally measured LODs.

The strongest translational evidence in this section was associated with designs that incorporated real biological matrices, internal referencing, multiplexed detection, or integrated sample handling, independently of transduction modality. Device architecture can improve sensitivity, multiplexing, and usability, but it does not by itself establish clinical relevance. Translation depends on whether the sensing strategy remains robust in the intended biological matrix, whether performance is reproducible across devices and batches, and whether the output can be compared with an established clinical method. Table 3 summarizes the main transduction platforms, material architectures, strengths, and limitations identified across the 18 Section 5 studies.

## 6. Biomedical Targets and Biological Matrices

**Figure 9:**
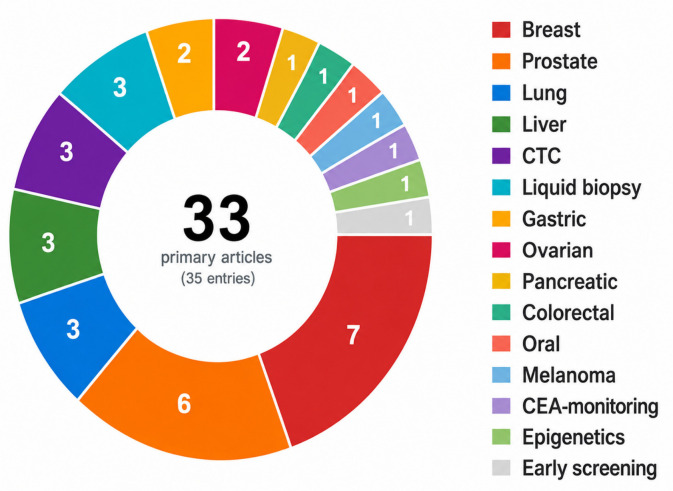
Distribution of cancer indications across the 33 Section 6 primary experimental publications. Breast cancer was the most frequent indication, followed by a long tail of indication-specific platforms. Bar height represents the number of unique articles per indication; biosensor-level analytical entries are charted separately in Supplementary Material S3, Section 6 charting.

This section maps cancer indications, biomarker targets, recognition strategies, transduction formats, and analytical performance across 33 primary experimental publications retained from the 2024–2026 study set. Because two publications reported analytically distinct biosensor platforms, the Section 6 evidence map comprises 35 biosensor-level analytical entries. The studies addressed 14 cancer types and more than 20 biomarker targets, illustrating the versatility of nano-carbon biosensors while also revealing a fragmented landscape; rather than converging around clinically prioritized biomarkers or use cases, recent work continues to explore heterogeneous targets, matrices, and device formats (Figure 9).

### 6.1. Nano-Carbon Materials and Biomarker Distribution

Graphene and its derivatives were the most frequent nano-carbon class, appearing in approximately two-thirds of the analytical entries, reflecting their favorable charge-carrier mobility and chemically accessible surfaces. Bare graphene served primarily as an active electronic channel in transistor-based platforms for detecting miRNA, RNA, protein biomarkers, ctDNA, and circulating tumor cells [57,58,59,60,61]. rGO and GO functioned as electrochemical, resistive, or signal-modulating components for multiplex miRNA detection, PCA3 RNA sensing, exosome detection, and dual-channel CA-125 aptasensing [30,62,63,64,65]. Other graphene-derived materials, GQDs, carboxylated graphene, tree-like graphene, and LIG, expanded the family toward PSA immunosensing, Annexin A2 detection, cancer-cell morphology sensing, epigenetic detection, and multiplexed electrochemical formats [22,66,67,68,69].

Carbon nanotubes formed the second major material category. SWCNTs were used in electronic or sequence-specific sensing platforms for PSA, KRAS G12D ctDNA, and amplification-free ctDNA detection in plasma [70,71,72]. MWCNTs served as electrode modifiers or conductive scaffolds for hydrogen peroxide monitoring, EpCAM-positive cell detection, and Claudin18.2 immunosensing [73,74,75,76].

Carbon dots and CQDs were applied in optical and electrochemiluminescent formats for miRNA detection, hepatocellular carcinoma biomarker sensing, bimodal PSA detection, and gene detection in saliva-like matrices [77,78,79,80,81]. A reporting gap worth noting: the nano-carbon component was incompletely characterized in three studies [82,83,84,85], limiting reproducibility assessment, as material identity, oxidation state, and surface chemistry directly affect signal generation and biofunctionalization.

### 6.2. Cancer Types and Biomarkers Investigated

The 33 primary experimental publications, corresponding to 35 biosensor-level analytical entries, covered 14 cancer types, with breast, prostate, lung, and liver cancer most frequently represented. Breast cancer studies targeted microRNAs, exosomes, and serum-based molecular markers through graphene transistor platforms, dual-miRNA sensing, and exosome-based detection [61,62,65,81,85]. Prostate cancer was addressed through PSA detection, PCA3 RNA sensing, and epigenetic 5hmC analysis [22,64,68,70,78]. Lung cancer studies included multiplex microRNA detection, simultaneous protein-marker detection, and machine-learning-assisted classification [30,59,66]. Liver cancer was assessed using Annexin A2 detection, AFP aptasensing and 5hmC analysis [67,68,77].

**Figure 10:**
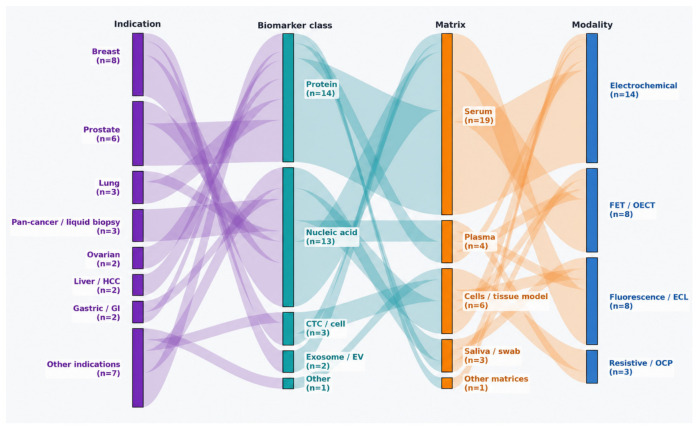
Sankey diagram linking cancer indication, biomarker class, biological matrix, and transduction modality across Section 6 included studies (33 primary publications; 35 biosensor-level analytical entries). Full flow-level charting is provided in Supplementary Material S3, Section 6 charting.

The biomarker landscape was highly heterogeneous. Protein targets included PSA, CEA, AFP, NSE, CYFRA21-1, CA-125, CA 15-3, EpCAM, Claudin18.2, Annexin A2, Vimentin, p53, SOX2, and lactate dehydrogenase. Nucleic-acid targets included multiple microRNAs, ctDNA mutations, PCA3 RNA, and Helicobacter pylori genes. Exosomes, hydrogen peroxide, and 5hmC further expanded the dataset to encompass liquid biopsy, tumor-microenvironment monitoring, and epigenetic detection. Multiplexed designs were a clinically relevant feature of several entries, including four-miRNA panels, simultaneous detection of lung-cancer protein markers, dual-miRNA sensing, and dual-antibody cancer-cell identification [30,59,66,75,81]. Figure 10 integrates cancer indication, biomarker class, biological matrix, and transduction modality in a Sankey representation.

### 6.3. Recognition Elements and Functionalization Strategies

Antibodies were the dominant bioreceptors, used in immunosensors for protein biomarkers and circulating tumor cells targeting EpCAM, CEA, PSA, Claudin18.2, and multiplex protein panels [22,58,59,66,73,75,78,82]. As described, immobilization involved linker-mediated anchoring, carbodiimide coupling, antibody–enzyme bioconjugation, or protein blocking, but the chemical details were inconsistent across entries. Aptamers were used for AFP, CA-125, and RNA-associated analytes on carbon dot, GO, or graphene transistor platforms [61,63,77]. DNA-based recognition was applied to nucleic-acid biomarkers using hairpin probes, guide-DNA complexes, complementary ssDNA, and sequence-specific ctDNA probes [30,57,64,72,81]. Less conventional mechanisms included molecular-corona adsorption for ctDNA discrimination, PSA-affinity peptides, and G-quadruplex/DNAzyme-based intracellular sensing [70,71,75].

**Figure 11:**
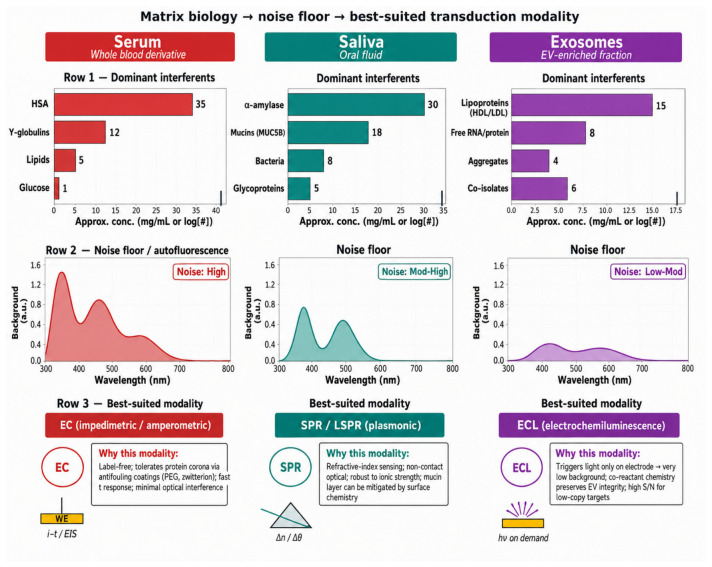
Matrix-specific constraints shaping nano-carbon biosensor design. Dominant interferents and baseline noise in serum, saliva, and exosome-enriched matrices influence the selection of recognition strategy, antifouling architecture, and transduction modality.

A critical reporting gap was the absence of clearly described recognition elements and/or immobilization chemistry in six biosensor-level entries [62,67,68,84,85,86]. This omission limits reproducibility because bioreceptor identity, orientation, density, and coupling efficiency directly affect specificity, stability, and method transfer. Figure 11 illustrates that biosensor design is shaped not only by cancer indication or biomarker class but also by the biological matrix in which detection is intended to occur.

### 6.4. Biosensing Platforms and Transduction Methods

Electrochemical platforms dominated, spanning label-free immunosensors, sandwich immunoassays, chemiresistive sensors, paper-based devices, cancer-cell sensors, and one implantable microelectrode. FET architectures formed an important electrochemical subgroup, particularly in graphene-based devices; flexible, CRISPR-Cas10-integrated, solution-gated, vertical, Argonaute-mediated, rGO array and organic electrochemical transistor formats addressed RNA/miRNA detection, protein sensing, ctDNA discrimination, CTC detection and cancer-cell morphology monitoring [30,57,58,59,60,61,69,87,88]. Other electrochemical readouts included DPV, chronoamperometry, resistance change, and open-circuit potential monitoring [64,67,68,73,84].

Fluorescence and ECL formed the second major group. Fluorescence platforms included NIR aptasensing, smartphone-assisted digital fluorescence, ratiometric sensing, and intracellular probe delivery [75,77,79,80]. ECL was represented by carbon-dot platforms for detecting miRNA and CA-125 [63,81]. Two studies stood out for extending architecture beyond conventional ex vivo assays: Lin et al. [74] developed an implantable microelectrode for real-time hydrogen peroxide monitoring in solid tumors; Cai et al. [66] combined multiplexed LIG immunosensing with imaging and clinical variables in a machine-learning classifier. Flexible substrates, paper-based formats, wireless readout, and smartphone integration signal a field moving toward miniaturized, point-of-care-compatible systems.

### 6.5. Analytical Performance

Per-device analytical performance data for the Section 6 publication-level records, with platform-level distinctions where available, comprise numeric LOD and analyzed/linear concentration range (with units), target biomarker, biological matrix, and transduction modality, and are compiled in Supplementary Material S3. Records for which the primary study did not report a working/linear range are marked NR in that table, as omission of this parameter is itself a reporting-gap finding (see also Section 7.1). Analytical sensitivity across the biosensor-level entries spanned more than ten orders of magnitude, from sub-femtomolar FET platforms to micromolar metabolite monitors, reflecting the diversity of recognition strategies, transduction modalities, and target classes. The strongest analytical performance was reported by FET platforms [59,67,77], ECL dual-miRNA platforms for triple-negative breast cancer [81], and carbon-dot-based fluorescence/electrochemical PSA platforms [78]. Multiplexed LIG immunosensors demonstrated high analytical performance across multiple lung cancer biomarkers [66].

Beyond LOD, the Section 6 publication-level records (*n* = 33) present an uneven reporting landscape for the parameters that most directly determine clinical usability. Recovery in real matrix was reported in 12 of 33 records (36%); intra-assay precision (CV%) in 16 (48%); selectivity against named clinically relevant interferents in 23 (70%); analyzed/linear concentration range in 29 (88%); response time in 22 (67%); and formal comparator against a reference method in 15 (45%). Real sample validation of any kind was reported in 30 of 33 records (91%), but most used spiked matrices rather than confirmed patient specimens. LOD and linear range have the highest reporting coverage; recovery, inter-batch precision, and interferent robustness are the least reported—precisely the parameter set most relevant to translational readiness. This reporting asymmetry is quantified in the minimum reporting set proposed in Supplementary Material S2.1 and feeds directly into Bottleneck 2 (Section 7.1).

### 6.6. Sample Type and Validation Level

The biological matrices ranged from buffer-only proof-of-concept measurements to spiked human serum and limited patient cohorts. Serum and plasma dominated, used across the majority of entries for protein markers, ctDNA, miRNA panels, and exosome-based detection. Saliva was used for Claudin18.2 immunosensing [73], smartphone-based protein detection [80], and in vivo monitoring of hydrogen peroxide [74]. Urine supported PCA3 detection [64]. Intracellular, in vivo, and tumor-microenvironment matrices were represented by the artificial liposomal cell platform [75] and the implantable microelectrode [74].

Validation depth varied substantially across the 33 publication-level records and 35 biosensor-level analytical entries. The counts below refer to the Section 6 records available in Supplementary Material S3. Two records used buffer or synthetic saliva only. Approximately 12 records reported performance in spiked human matrix without a formal clinical comparator. Six records tested clinical samples from confirmed patients without a reference method comparison. Fifteen records included a formal clinical comparator—ELISA, chemiluminescence immunoassay, qPCR, or equivalent—with reported agreement. A consolidated map of validation level by record, categorized as buffer-only, spiked matrix, patient samples without comparator, and patient samples with formal comparator, is provided in Supplementary Material S3, Section 6 charting. The aggregate numbers are cited in Section 7.2, where the validation gap is developed as Bottleneck 1.

## 7. Translational Barriers and Technological Maturation

**Figure 12:**
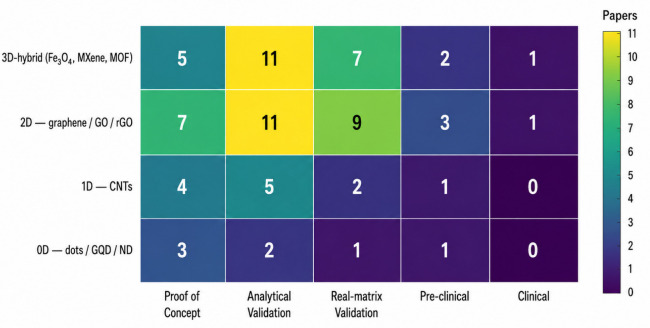
Translational maturity distribution across the Section 7 working set. Studies cluster predominantly at the Analytical and Real-matrix TRL stages across nano-carbon families, whereas Preclinical and Clinical-stage evidence remains sparse regardless of material geometry. Per-study TRL assignments are provided in Supplementary Material S3, Section 7 charting.

Across the 188 charted primary studies, the Evidence Level distribution was dominated by Low-evidence reports (125/188, 66.5%), followed by Intermediate-evidence (45/188, 23.9%) and Strong-evidence studies (18/188, 9.6%) (Supplementary Material S3, Section 7 charting). On the TRL-inspired axis, 41% of studies were classified as Analytical Validation, 27% as Real-matrix Validation, 22% as Proof of Concept, 8% as Preclinical, and 2% as Clinical. The two densest cells of the Translational Readiness Matrix were Intermediate/Analytical and Intermediate/Real-matrix, whereas the sparsest were Strong/Preclinical and Strong/Clinical, with the latter populated by fewer than ten studies. Within the Section 7 working set of 87 studies, 25 studies (28.7%) reported data in real clinical samples, but only eight (9.2%) included a formal comparator against a clinical reference method with an agreement metric; the bottleneck developed in Section 7.2. Figure 12 maps this maturity distribution, showing the concentration of studies at the Analytical and Real-matrix TRL stages across graphene-family and composite architectures. These distributions provide the quantitative foundation for the translational and regulatory assessments in Section 7 and Section 8; the 188-study Evidence Level and TRL-axis distributions underpin the Translational Readiness Matrix cell assignments, while the 87-study full-text counts ground the bottleneck characterization and the formal comparator-agreement gap reported throughout.

Two-dimensional carbons sit at the center of this working set. Twenty-one studies work with graphene, GO or rGO alone; a further 14 combine these sheets with three-dimensional hybrid components such as Fe_3_O_4_, MXene or MOF particles. Carbon nanotubes appear in 18 studies, zero-dimensional carbons in seven, and MIP composites in nine. Transduction is overwhelmingly electrochemical: approximately 40 designs use voltametric, amperometric or impedimetric readouts; 14 employ FETs; 13 use optical or ECL transduction; and only four fall into the wearable point-of-care category.

Of the 87 studies included in this section, 60 (68.9%) were classified as Real-matrix, meaning they were tested in clinically relevant biological specimens, most often serum, but without meeting the criteria for robust clinical validation. A further 18 studies (20.7%) were Proof-of-concept, evaluated under controlled analytical conditions with limited translational projection. Two studies (2.3%) provided Preclinical in vivo evidence restricted to animal models. Only seven (8.0%) reached the Real-matrix/Clinical category, that is, evaluation in confirmed patient specimens against an identifiable diagnostic comparator. This distribution points to a persistent translational gap; strong analytical performance in real matrices, while necessary, is not on its own sufficient evidence of clinical readiness, and independent validation cohorts, diagnostic comparators, inter-batch reproducibility, and workflow integration remain largely unaddressed across the corpus.

Among the biosensor platforms identified, immunosensors were the most frequent architecture (*n* = 23), followed by electrochemical biosensors (*n* = 16), aptasensors (*n* = 14), and field-effect transistor-based devices (*n* = 7). This distribution reflects a predominance of molecular recognition-based strategies, and electrochemical transduction emerged as the most recurrent detection principle across platform types. This pattern is consistent with the field’s orientation toward miniaturizable, low-cost, and point-of-care-compatible devices. Platform diversity alone, however, does not resolve the translational gap described above; regardless of architecture, most of these devices were evaluated at the Real-matrix stage, and Clinical-grade evidence remained scarce across all biosensor categories.

Serum was the predominant biological matrix across the dataset (*n* = 45), followed by in vivo specimens (*n* = 6) and human serum (*n* = 5). The categorical distinction between “serum” and “human serum” as recorded across studies reflects an underlying inconsistency in matrix reporting, which limits direct cross-study comparability and suggests the absence of a unified nomenclature standard in the field. Taken together, serum-based evaluations account for the vast majority of sample types, reinforcing the pattern seen in the translational readiness and biosensor-type analyses; platforms are mostly developed and tested within a single, accessible biological matrix, with little systematic exploration of alternative specimen types such as whole blood, urine, or saliva, which would be more compatible with minimally invasive or point-of-care deployment. This matrix convergence, while analytically pragmatic, further narrows the evidence base available to support the broader clinical applicability of the platforms described.

Reported limits of detection across the 87 included studies span several orders of magnitude, from sub-attomolar values in nucleic acid-targeting platforms to low nanogram-per-milliliter thresholds in protein immunosensors. This range reflects the heterogeneity of biomarker classes, transduction methods, and matrix conditions rather than a coherent performance benchmark. Linear detection ranges were similarly variable and frequently non-overlapping across studies, and sensitivity, when reported, was expressed in inconsistent formats across platforms, precluding direct aggregation. Collectively, these parameters confirm the analytical viability of the platforms under their respective experimental conditions; however, the absence of standardized reporting units, comparator methods, and inter-laboratory reproducibility data renders cross-study performance comparisons methodologically unsound. Accordingly, a low LOD and a broad linear range, while indicators of platform sensitivity, should be interpreted as necessary but insufficient criteria for translational readiness, a conclusion reinforced by the scarcity of formal clinical comparators and validated patient cohorts documented in this corpus.

### 7.1. Five Recurring Bottlenecks

**Figure 13:**
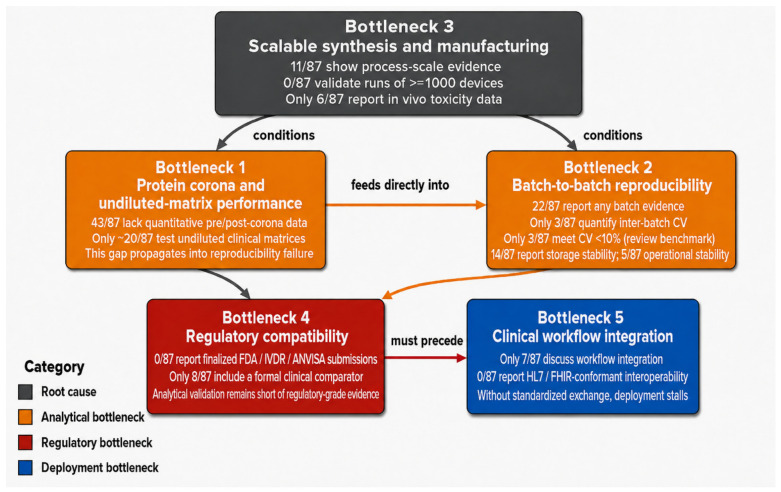
The five recurring translational bottlenecks form an interconnected causal chain rather than an independent list. Scalable synthesis (Bottleneck 3, gray) conditions both protein-corona control and performance in undiluted matrices (Bottleneck 1, orange), as well as batch-to-batch reproducibility (Bottleneck 2, orange). These analytical bottlenecks constrain regulatory compatibility (Bottleneck 4, red) and, in turn, the integration of biosensors into clinical workflows (Bottleneck 5, blue). Numerical labels within each node summarize the main reported evidence gap.

Across families and modalities, the same five constraints surface repeatedly, and they are not independent of one another. Figure 13 maps how they interlock, tracing the translational pathway from the molecular interface through device manufacturing to the clinical workstation; each gap inherits unresolved issues from the preceding one.

Bottleneck 1: Protein corona and undiluted-matrix performance. The first gap sits at the biological interface. Only 44 of the 87 working-set studies provided quantitative characterization of the protein corona before and after matrix exposure, leaving the antifouling picture incomplete for nearly half of the field. Most groups relied on spiked serum, and only approximately 20 studies benchmarked their platforms against undiluted clinical matrices. Abundant serum proteins such as albumin, fibrinogen, and immunoglobulins can adsorb competitively onto the biointerface within seconds, altering the effective association constant and degrading analytical performance in ways that buffer-spiked experiments cannot capture. This shortcoming feeds directly into Bottleneck 2.

Bottleneck 2: Batch-to-batch reproducibility. Although 22 of the 87 studies reported some form of batch-reproducibility information, only three quantified inter-batch coefficients of variation. The remaining 19 studies mentioned batch behavior without providing a quantitative inter-batch CV, while 65 studies did not mention batch reproducibility at all. Only three studies achieved inter-batch CV ≤ 10%, a conservative reproducibility benchmark adopted in this review rather than a universal FDA threshold. Current FDA/ICH bioanalytical guidance generally uses precision limits of ≤15% CV, except at the LLOQ, where ≤20% CV is allowed. Shelf-life and operational stability were also sparsely reported; 14 of the 87 studies reported storage-stability data, whereas stability under operational conditions, such as repeated use, temperature cycling, or humidity variation, was reported in only five studies. Thermal stress was evaluated in one study, and mechanical stress in none of the included studies. A platform that cannot demonstrate both inter-batch reproducibility and operational stability under intended-use conditions lacks the minimum manufacturing evidence needed for translational assessment, regardless of its analytical sensitivity. Table 4 summarizes this attrition pattern, from no batch reporting to qualitative mention, quantified inter-batch CV, conservative CV compliance, real-clinical-sample testing, and formal comparator-based validation.

Bottleneck 3: Scalable synthesis and manufacturing. Scalable production of nano-carbon biosensors remains largely a laboratory-stage exercise. Only 11 studies presented process-scale evidence, and none validated production runs of ≥1000 devices, leaving unresolved issues of cost, batch consistency, quality control, and regulatory compatibility. Scalability operates at three levels. First, nano-carbon synthesis can, in principle, be scaled via CVD, CCVD, hydrothermal, or chemical routes, but transferring functional specifications from laboratory batches to production remains the main quality-control bottleneck. Second, device fabrication imposes architecture-specific constraints: screen-printed electrodes are compatible with low-cost, high-throughput manufacturing, whereas FET-based platforms require more complex semiconductor-compatible fabrication, alignment, and packaging. Third, clinical implementation requires more than device fabrication alone; it also entails cold-chain independence, user training, hospital information system integration, quality assurance procedures, and reimbursement pathways, which were rarely addressed in the included studies.

Evidence of safety and biocompatibility remains similarly underdeveloped. The working set reported in vivo toxicity data in only six studies, which should be interpreted as a reporting gap rather than evidence of safety. Family-specific considerations are relevant: high-aspect-ratio MWCNTs raise pulmonary-hazard concerns [89,90], although this risk is substantially reduced when nanotubes are shortened, embedded, or substrate-immobilized, as in most biosensor formats; graphene and GO show dose-, size-, oxidation-, and route-dependent toxicity profiles [91]; CDs and GQDs are generally positioned as lower-toxicity nano-carbon options; and iron-oxide cores in magnetic–carbon hybrids are commonly used as comparatively biocompatible magnetic phases. Together, these gaps show that scalable manufacturing cannot be evaluated in isolation from material specification, device architecture, stability testing, and safety documentation.

Bottleneck 4: Regulatory pathway. No primary study in the Section 7 working set reported a finalized regulatory submission to the FDA, an EU IVDR Notified Body, or ANVISA (0/87 studies). For context, only eight of the 87 studies included a formal comparator against a clinical reference method with a defined agreement metric, underscoring the distance between analytical validation and regulatory-grade evidence (see Section 7.1 and Section 7.4). The absence of reported regulatory engagement suggests that the field remains predominantly within an exploratory research regime, with certification planning rarely integrated into biosensor development.

Bottleneck 5: Integration with clinical diagnostic workflows. Workflow integration was reported in only seven of the 87 studies (8.0%), and none demonstrated conformance with HL7/FHIR or equivalent interoperability standards for electronic health-record integration. Without standardized data exchange, an analytically precise biosensor remains an isolated instrument, unable to enter the clinical decision loop. This is the most downstream bottleneck, but it conditions translational success as fundamentally as the upstream ones; a device that cannot communicate its output to clinical information systems is unlikely to change clinical practice, regardless of its analytical sensitivity or regulatory status. Figure 13 maps the five recurring bottlenecks as a translational chain from nano-carbon synthesis and biointerface performance to regulatory compatibility and clinical workflow integration.

### 7.2. Exemplars from the Upper Bound of the Included Studies

**Figure 14:**
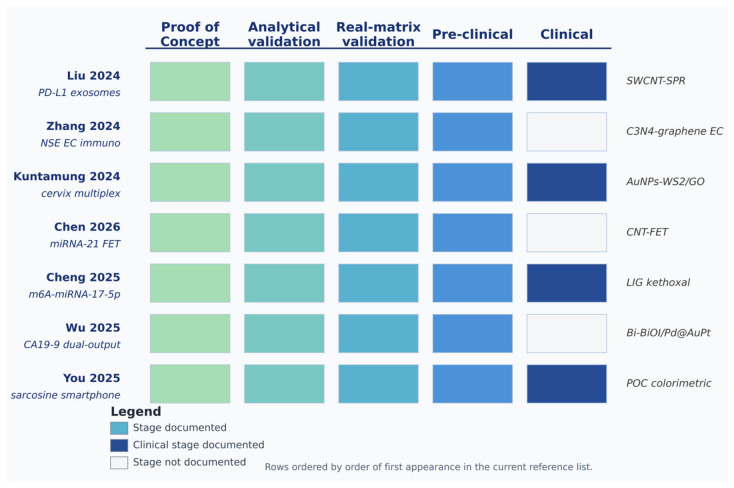
Translational trajectory of the eight upper-bound Real-matrix/Clinical exemplar platforms [28,55,59,102,103,104,105]. Each horizontal lane corresponds to one exemplar, with milestones positioned at the furthest validation tier reached by that platform. The shared pattern is progression through analytical and Real-matrix Validation toward early clinical-pilot evidence, without demonstration of manufacturing-scale reproducibility or finalized regulatory submission. Per-study details are provided in Supplementary Material S3, Section 7 charting.

Table 5 summarizes these upper-bound exemplars by cancer type, biomarker, biological matrix, transducer architecture, clinical cohort size, comparator method, and LOD. Eight studies occupy the Real-matrix/Clinical tier of the Translational Readiness Matrix, representing the upper bound of clinical evidence identified in the Section 7 working set (Figure 14). Each was evaluated in confirmed human clinical specimens—blood, serum, plasma, or whole blood from cancer patients—and collectively covers a range of biomarker classes and cancer indications. The targets span multiplexed lung-cancer protein panels, protease activity signatures in lung cancer, EpCAM-positive cells in metastatic lung cancer, oncoprotein c-Myc in lymphoma, extracellular-vesicle miRNAs in liver cancer, PD-L1-positive CTCs in NSCLC, circulating tumor cells, and piRNA-54265 in colorectal cancer [92,93,94,95,96,97,98].

Four further studies, covering CTC detection [98], urinary biomarkers [99,100], and implantable wireless sensing [101] or smartphone-readable point-of-care formats [102], extend the translational discussion without yet meeting all Strong–Clinical criteria; full per-paper details appear in Supplementary Material S3, Section 6 charting. The lesson cutting across this upper-bound set is that clinical evidence in the nano-carbon biosensor literature remains nascent even at the Real-matrix/Clinical tier. Cai and Dempsey et al. [66,92] report formal comparator-anchored performance metrics, and this remains the strongest clinical-validation exemplar because it reports quantified sensitivity and specificity across the largest cohort (*n* = 750 assays; sensitivity 90%, specificity 82%). Liu YP and Saputra et al. [40,86] document confirmed patient specimens but without a formal reference-method comparator. The remaining four studies report clinical matrix evaluation without comparator agreement, batch reproducibility, or stability data. These platforms should therefore be read as early translational evidence rather than validated clinical diagnostics. Three observations emerge from this upper-bound set.

A quantitative characterization of clinical cohort size across the 25 studies that report data in real human clinical samples reveals a distribution concentrated at the lower end of statistical defensibility. Cohort sizes range from small patient groups to a maximum of 750 assays across 450 unique donors [92]. Within the eight Real-matrix/Clinical exemplars, clearly reported cohort sizes remain limited or incompletely specified in several studies, with Dempsey and Cai et al. [66,92] standing out as the most developed Clinical-cohort examples. This distribution has a direct methodological implication; studies with *n* < 30 per group generally cannot estimate sensitivity and specificity with 95% confidence intervals narrow enough to support a robust clinical claim, regardless of the point estimate. The field’s clinical validation evidence base is therefore not only sparse in coverage but also frequently underpowered, which compounds the formal comparator gap (eight of 87 studies, 9.2%) identified in Section 7.1.

Matrix conditioning as a translational discipline. The strongest devices condition the sample before transduction rather than relying on raw sensitivity. The sub-attomolar and low-nanomolar LODs recorded in Table 5 across the Real-matrix/Clinical platforms reflect the analytical headroom required to detect low-abundance targets in complex biological specimens. Platforms such as Liu and Yu et al. [95,97] achieve attomolar sensitivity in clinical EVs and diluted serum, respectively, demonstrating that matrix-compatible detection is technically feasible. However, the translational implication is that LOD is a necessary input to the matrix-conditioning design, not the property that earns clinical credibility on its own.

Clinical specificity and biomarker selectivity. The second observation concerns how Real-matrix/Clinical platforms handle competing signals in complex specimens. Liu YP et al. [45] simultaneously capture CTCs and monitor PD-L1 expression in NSCLC patient blood, combining two clinically relevant endpoints in a single liquid-biopsy workflow. Cai et al. [66] integrate a multiplexed lung-cancer biomarker panel with machine-learning-supported classification, while Dempsey et al. [92] discriminate lung cancer patients from cancer-free controls through enzymatic protease activity signatures rather than protein concentration alone. Each additional analytical dimension multiplies failure modes; the studies that reach Real-matrix/Clinical placement are those that demonstrate selectivity in patient specimens rather than in buffer alone.

Comparator agreement as the translational bottleneck. The third observation concerns the most consistent gap in the Real-matrix/Clinical set. Cai and Dempsey et al. [66,92] report comparator-anchored performance metrics, but the remaining six studies evaluate performance in patient specimens without benchmarking against an accepted clinical reference method with quantified agreement. This pattern confirms that testing in clinical samples is a necessary but insufficient step toward clinical utility; without comparator-anchored validation, the diagnostic value of the platform remains undetermined regardless of the biological matrix used.

Read together, these three observations suggest that Real-matrix/Clinical placement reflects the use of patient specimens rather than the completion of a full validation envelope. Matrix-compatible detection, clinically relevant biomarker selection, adequate cohort size, and, where available, comparator-anchored evaluation are the practices that move a device from analytical demonstration toward a credible clinical pilot, and they are the practices that most of the working set has yet to implement.

### 7.3. Central Observation

The quantitative evidence from the charted studies shows a consistent pattern of technical maturity with translational constraint. Many Section 7 studies report LODs within or below clinically relevant concentration windows, indicating substantial analytical maturity at the detection level. However, only three of 87 studies (3.4%) reported inter-batch CVs ≤ 10%, only 25 of 87 (28.7%) reported data from real human clinical samples, only eight of 87 (9.2%) included a clinical reference comparator with a defined agreement metric, and none reported finalized regulatory engagement with FDA, IVDR, or ANVISA pathways. Together, these figures show that the field can often detect relevant targets but has not yet demonstrated manufacturability, clinical agreement, workflow integration, or regulatory readiness at scale.

The central observation can be stated plainly: a low limit of detection is necessary but not sufficient for clinical utility. The upper-bound Real-matrix/Clinical exemplars summarized in Table 5 are not simply the studies with the lowest LODs. Rather, they are the studies that combine acceptable analytical sensitivity with patient–specimen testing and, in the strongest cases, comparator-anchored performance evaluation. Low LOD remains essential for applications such as ctDNA detection, exosomal mutation panels, and early CTC capture, but across the mapped design space, analytical sensitivity alone does not predict clinical trajectory.

This synthesis leads to four minimum reporting priorities. First, studies should report quantitative matrix-effect characterization, including recovery, interference, and matrix-induced signal suppression or enhancement. Second, inter-batch CV should be measured across at least five independent fabrications. Third, stability should be reported under storage and operational conditions relevant to intended use. Fourth, comparator-grade validation should be performed against a clinical reference method or accepted diagnostic framework, using an appropriate agreement metric such as Bland–Altman analysis, Passing–Bablok regression, kappa, ROC/AUC, sensitivity/specificity, or equivalent measures.

The complete nine-item minimum reporting checklist, including the six core analytical-reporting items and the three claim-dependent sovereignty/deployment items, is provided in Supplementary Material S2.1. Overall, the mapping patterns developed across Section 7.1, Section 7.2 and Section 7.3 address RQ3: translational maturity is unevenly distributed across the nano-carbon biosensor design space. Only approximately 10% of the 191-study evidence map reaches the Strong Evidence Level, whereas approximately 67% remains anchored in the Low Evidence Level. The actionable path forward is therefore not simply to lower LODs further but to improve reproducibility, Real-matrix Validation, comparator agreement, manufacturing evidence, regulatory planning, workflow integration, and clinical cohort design.

### 7.4. Empirical Anchoring Against the Clinical Trials Registry

An independent ClinicalTrials.gov registry scan conducted in May 2026 identified registered cancer studies involving carbon nanoparticle technologies, but the distribution differed sharply from the biosensor literature mapped in this review. Most registered studies involved carbon nanoparticle suspension as an intra-operative tracer for lymph-node mapping or surgical guidance, including colorectal cancer and breast cancer protocols such as NCT06783985, NCT04482803, and NCT07032220. A smaller group involved diagnostic or contrast-agent applications, including NCT07295340, a prospective observational ex vivo study evaluating folate-targeted NIR-II carbon dots for distinguishing hepatocellular carcinoma tissue from normal liver tissue. One Phase 1 therapeutic study, NCT06048367, evaluated intratumoral carbon nanoparticle-loaded iron, CNSI-Fe(II), in patients with advanced solid tumors.

Mapping these registered studies to the TRL axis yields a distribution that contrasts with the published biosensor evidence base. In registered clinical evaluation, cancer nano-carbon technology has matured primarily as surgical tracer, contrast-agent, or therapeutic platforms rather than as electrochemical, FET, optical, or wearable biosensor diagnostics. Within the registry scan, no identified trial corresponded directly to the biosensor architectures that dominate the analytical literature examined in this review.

This inversion reinforces the central observation of Section 7.4: analytical sensitivity alone does not predict translational progress. The technologies that have advanced into registered clinical evaluation are not necessarily those reporting the lowest LODs under buffer or spiked-matrix conditions but those with a clearer intended use, clinical workflow fit, and pathway toward patient-facing evaluation. The registry therefore provides an external referent for the Translational Readiness Matrix, showing that movement toward clinical deployment depends on more than analytical performance.

These findings also address RQ2. The analytical performance envelope—LOD, dynamic range, reproducibility, and matrix recovery—is matrix-conditioned. Serum, saliva, urine, blood, and exosome-enriched samples impose distinct interferent and noise profiles that constrain modality choice independently of the biomarker target. This matrix-conditioning challenge helps explain the gap between the published nano-carbon biosensor literature and the registered clinical-trial landscape.

## 8. Technological Sovereignty and Strategic Autonomy in Nano-Carbon Biosensor Ecosystems

The preceding sections examined nano-carbon biosensors from the inside out: material design (Section 3), biointerface chemistry (Section 4), transduction strategy (Section 5), biomedical targets and matrices (Section 6), and translational barriers (Section 7). Section 8 turns the lens outward and asks what translation requires from the system within which a biosensor is developed, certified, manufactured, and deployed. The evaluation lens draws on two converging frameworks, the EUREL biomedical-engineering sovereignty model, which maps sovereign capacity across the technology lifecycle [106], and the European Commission strategic-dependencies framework, which classifies dependencies by criticality and concentration [107]. Throughout this section, technological sovereignty denotes the capacity of a country or jurisdiction to develop, manufacture, certify, and deploy a technology with sufficient independence from external choke points; the term is not used in a geopolitical or value-laden sense.

Across the analytical dimensions examined in this review, nano-carbon biosensors have demonstrated substantial analytical maturity, including sub-femtomolar LODs, multimodal architectures, and increasing Real-matrix Validation. However, this analytical maturity is not matched by equivalent translational maturity in regulatory submission, scaled manufacturing, or jurisdiction-specific deployment. The transition from analytical performance to translational sovereignty therefore requires that a device be evaluated against research, production, regulatory, and deployment capacities, not only against its LOD or transduction signal.

### 8.1. Supply-Chain Concentration and the Limits of Binary Governance

China accounts for approximately 78% of global natural graphite mine production [108]; export-license dynamics since 2023 [109] introduce supply chain volatility for graphite-derived nanocarbons, graphene oxide, rGO, and GQDs. Binary dual-use classification is ill-suited to enabling technologies with indistinguishable civilian and security-relevant end-uses. Walker-Munro [110], using quantum technology as a limiting case, advocates a gradient-based sovereignty taxonomy that places biomedical applications, biosensors, theranostics, and implantable diagnostics in a regulated-civilian category whose risk profile is distinct from defense-strategic applications. For nano-carbon biosensors specifically, this distinction matters because supply-chain disruption does not affect military and diagnostic end-uses symmetrically; a shortage of high-purity graphite affects surgical-grade implantables and point-of-care diagnostics before it affects most defense applications, making the regulated-civilian category the more urgent locus of sovereignty planning.

### 8.2. The Bench-to-Market Sovereignty Gap: Regulatory Capacity and Translational Infrastructure

Supply-chain concentration is the first sovereignty constraint for nano-carbon biosensor ecosystems. China accounts for approximately 78% of global natural graphite mine production, creating an upstream dependency for graphite-derived nanocarbons, including graphene oxide, rGO, GQDs, and related carbon precursors. Export-license dynamics introduced after 2023 further illustrate how access to high-purity graphite and processed nano-carbon inputs can become a bottleneck for biomedical research, prototype development, and future diagnostic manufacturing.

This dependency is difficult to manage through binary dual-use governance. Nano-carbon materials are enabling platforms; the same precursor, synthesis route, or surface chemistry may support civilian diagnostics, surgical tracers, implantable sensors, environmental monitoring, or security-relevant applications. For this reason, a gradient-based governance model is more appropriate than a simple civilian-versus-defense classification. In the context of this review, nano-carbon cancer biosensors fall primarily within a regulated-civilian category; their strategic relevance arises not from weaponization but from their potential role in diagnostic autonomy, clinical resilience, and health-system preparedness.

**Figure 15:**
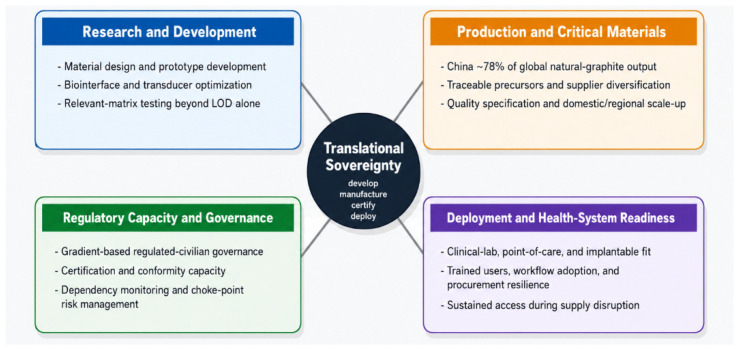
The central finding illustrated is that sustained clinical utility of nano-carbon biosensors requires concurrent capacity across four domains and that a deficiency in any one domain constitutes a sovereignty bottleneck regardless of analytical performance in the others.

For biosensors specifically, supply-chain disruption would not affect all applications equally. Shortages of high-purity graphite or qualified nano-carbon inputs could delay laboratory prototyping, quality-controlled batch production, surgical-grade materials, and point-of-care diagnostic development long before a device reaches clinical deployment. Sovereignty planning for nano-carbon biosensors must therefore address material traceability, supplier diversification, quality specification, and domestic or regional manufacturing capacity as part of translational readiness, not as an external issue separate from biosensor design (Figure 15).

### 8.3. Evaluation Framework and Reporting Implications

The sovereignty-informed framework extends the Translational Readiness Matrix by adding a system-level layer to biosensor evaluation. The four domains used here, Research and Development, Production and Critical Materials, Regulatory and Data Governance, and Deployment, translate technological sovereignty into operational reporting criteria for nano-carbon biosensors.

Three additional reporting items follow from this layer. First, studies with translational intent should report the supplier, batch, and country of origin of critical upstream materials, including graphite, carbon precursors, functionalization reagents, and hybrid nanocomposite components where applicable. Second, they should declare the intended regulatory status or pathway, distinguishing research-use-only prototypes from devices positioned for FDA, IVDR, ANVISA, or equivalent routes, and should identify the remaining evidence gaps before submission. Third, point-of-care, wearable, connected, or lab-on-chip platforms should describe the intended data architecture and any planned interoperability strategy, including HL7/FHIR or equivalent standards where relevant.

Two commercial-readiness items should also be declared when deployment potential is claimed. Studies should provide at least a preliminary cost-of-goods or unit-cost estimate at a meaningful manufacturing scale, and they should state whether reimbursement, market access, or clinical-utility evidence would be required for the intended use case [111,112]. These items are not expected to be fully resolved in proof-of-concept studies, but their absence should be visible rather than hidden behind analytical performance.

The sovereignty-layer reporting items defined in Section 8.3 are incorporated into the nine-item checklist in Supplementary Material S2.1, and the per-study extraction data supporting the translational-readiness analysis are provided in Supplementary Material S3. These items complement the analytical reporting requirements developed in Section 7 and support checklist-based assessment of studies with translational claims.

## 9. Conclusions and Strategic Perspectives

The most direct practical contribution of this review is the nine-item minimum reporting checklist provided in Supplementary Material S2.1. Items 1–6 apply to all nano-carbon biosensor reports: material and precursor traceability; surface chemistry and biointerface construction; analytical performance with explicit definitions of LOD, LOQ, linear range, precision, and calibration model; matrix and protein-corona context; device-to-device and inter-batch reproducibility; and stability under intended storage and use conditions. Items 7–9 are claim-dependent and should be reported when relevant: biocompatibility and user-contact safety; regulatory positioning; and deployment, data architecture, and sovereignty-oriented reporting.

The Translational Readiness Matrix separates analytical achievement from clinical proximity. The resulting map is clear; only approximately 2% of the 191 primary studies reach the Clinical TRL bracket, and only approximately 10% reach the Strong Evidence Level. The central gap is therefore not the ability to produce ultralow LODs but the ability to document matrix effects, inter-batch reproducibility, stability, comparator agreement, manufacturing assumptions, and regulatory pathway. Across the mapped design space, analytical performance is shaped by the full biointerface chain, while progression toward clinical use depends on the research, manufacturing, regulatory, and deployment capacities described in Section 8.

### 9.1. Strategic Perspectives

Five strategic priorities emerge from this review.

Reporting standardization and checklist adoption. The checklist in Supplementary Material S2.1 should be treated as a methodological output of this review, not as a secondary appendix. Authors, reviewers, and editors can use it to assess whether studies with translational claims report the minimum information needed to judge reproducibility, matrix compatibility, stability, comparator validation, and deployment readiness.

Validation agenda. Real-matrix benchmarking against a formal clinical comparator remains the field’s most urgent unmet need. Future studies should move from isolated proof-of-concept claims toward validation packages that include recovery, reproducibility, Bland–Altman or equivalent agreement metrics, and cross-site confirmation when implementation is the stated goal.

Four-stage translational roadmap. Stage A should establish analytical validation in spiked matrix with at least five device replicates and four weeks of stability data. Stage B should test real clinical matrices in at least 30 clinical samples with a formal comparator and a pre-specified agreement metric. Stage C should add multisite reproducibility and pre-submission engagement for the intended regulatory pathway. Stage D should address regulatory submission, post-market surveillance planning, cost-of-goods modelling at the intended batch scale, and reimbursement or payer-coverage route.

Scalability, economics, and workflow readiness. Scalability must be evaluated at three levels: nano-carbon synthesis, device fabrication, and clinical deployment. Material cost is rarely the only constraint; antibodies, aptamers, manufacturing quality control, operator training, health-information-system integration, and reimbursement pathways may dominate the translational equation. A platform can be scalable at the material level and still fail at implementation if it cannot enter the intended diagnostic workflow.

Emerging frontiers with translational intent. AI-assisted decoding of transduction signals, implantable or intraoperative nano-carbon platforms, and closed-loop or wearable architectures deserve priority attention in the next biennium. These directions are promising only if paired with the same reporting and validation discipline required of electrochemical, FET, and optical systems.

### 9.2. Limitations of This Scoping Review

This review has limitations characteristic of the scoping methodology. The search was limited to six bibliographic databases and to English-language publications from 2024–2026; no formal critical appraisal of individual sources was performed; three metadata-only records were retained but could not be fully extracted at the cell level; and the focused sovereignty stream in Section 8 was assembled as a policy-analytic complement rather than as a standalone systematic review. These choices make the review useful for mapping the present landscape, but they also bound the strength and scope of the inferences that can be drawn.

The temporal restriction to the 2024–2026 biennium introduces a selection bias in two directions. Recent platforms benefit from improvements in nano-carbon synthesis, biofunctionalization, and AI-assisted signal interpretation, which may inflate apparent analytical maturity. At the same time, the restriction excludes earlier studies that established the translational foundations of the field, including early clinical-sample demonstrations, CNT scalability studies, and foundational protein-corona work. The result may be an illusion of analytical maturity; lower LODs are increasingly visible, while core translational bottlenecks remain structurally persistent.

The inclusion of theoretical and simulation-based studies also limits how broadly experimental conclusions can be generalized across families and modalities. Studies marked as theoretical or simulated in the evidence tables and figures remain valuable for design-space mapping, but they should not be interpreted as equivalent evidence for fabrication readiness or clinical translation.

Finally, the Translational Readiness Matrix is a new descriptive instrument developed for this review and was not formally validated before application. Its full inter-rater agreement was not calculated across the complete working set, its construct validity was not tested against downstream translational outcomes, and its numerical thresholds were not empirically calibrated for the nano-carbon biosensor literature. Future reviews should calculate full inter-rater agreement, test construct validity against registry-based outcomes such as ClinicalTrials.gov progression, and reassess the thresholds across broader time windows and disease areas.

### 9.3. Implications for Research, Practice, and Policy

Implications for research. Future protocol-level reporting should shift from isolated performance claims to integrated validation packages. Material selection, surface chemistry, matrix biology, and modality choice should be reported as an interdependent chain, and success should be judged less by record LOD claims than by comparator agreement, reproducibility [103], and matrix-appropriate performance.

Implications for clinical practice. The Clinical TRL bracket remains sparsely populated, which means that nano-carbon biosensors should currently be interpreted as adjunctive or triage-oriented tools rather than as stand-alone diagnostic replacements. Early deployment is most plausible in indications where biomarker panels and comparator methods are already established, and where the biosensor can enter a workflow that already has a clinical decision context.

Implications for policy and regulation. The Section 8 framework of technological sovereignty, understood here as the autonomous capacity to develop, manufacture, regulate, and deploy a diagnostic platform, places explicit demands on national and regional innovation systems. For Brazil and Latin America, supply-chain concentration in natural graphite, ANVISA pathway readiness, manufacturing quality systems, and HL7/FHIR-compatible data infrastructure emerge as linked constraints rather than independent issues.

Taken together, these implications argue for a shift from proof-of-concept publication logic to validation-oriented translational planning, in which analytical novelty, manufacturing realism, regulatory positioning, and health-system integration are treated as concurrent design variables rather than sequential afterthoughts.

### 9.4. Closing Statement

In the 2024–2026 biennium, nano-carbon biosensors for cancer produced a technically impressive but translationally uneven evidence base. The decisive test for the next cycle is no longer whether the field can report ever-lower LODs in buffer but whether it can routinely report matrix effects, inter-batch reproducibility, stability, comparator agreement, cost and manufacturing assumptions, regulatory pathway, and deployment context in a form that allows independent judgment of translational readiness. If those elements become standard, the field can move from analytically promising prototypes toward clinically credible platforms.

## Figures and Tables

**Table 1:** Comparative effects of covalent EDC/NHS coupling and pyrene-mediated π–π stacking on nano-carbon biointerface integrity, bioreceptor presentation, reproducibility, and electron-transfer behavior.

Criterion	Covalent EDC/NHS	π–π Stacking (PBSE/PyBSE)
sp^2^ network integrity	Compromised: –COOH groups required for activation introduce surface defects in the basal plane, reducing carrier mobility and field-effect sensitivity.	Preserved: adsorption on the intact basal plane leaves the π-conjugation network undisturbed, maintaining carrier mobility and transduction sensitivity at values characteristic of pristine material.
Bond stability	High: amide bond energy ~350 kJ/mol; stable across pH 4–9 and temperatures 4–40 °C; resistant to physiological ionic strength.	Moderate: π–π stacking energy ~20 kcal/mol per aromatic ring; susceptible to competitive displacement by endogenous aromatic molecules (bilirubin, phenylalanine, circulating nucleotides) in undiluted biological matrices.
Orientation control of bioreceptor	Partial: reaction with lysine ε-amines distributed across the antibody surface produces mixed orientations, increasing the risk of occluding the antigen-binding site and reducing effective recognition capacity.	High: pyrene-based linkers (PBSE, PyBSE) bear a reactive NHS terminus opposite the pyrene anchor, enabling oriented conjugation to primary amines while preserving antigen-binding site accessibility.
Inter-batch reproducibility	Low: coupling yield depends on –COOH surface density, which varies with nano-carbon supplier, oxidation method, and post-treatment. Two sets of studies using rGO from different suppliers reported a ~10-fold difference in coupling yield.	Moderate: adsorption efficiency depends on sp^2^ surface quality and specific area, variables that are more readily quantified by Raman spectroscopy than –COOH density, but still vary between synthesis batches.
Impact on electron-transfer kinetics	Variable: oxidation-induced defects and surface functional groups may create blocking sites that reduce the heterogeneous electron-transfer rate constant (k^0^) relative to unmodified carbon.	Positive: absence of covalent defects on the basal plane preserves the k^0^ values characteristic of pristine graphene or CNT surfaces, supporting higher exchange current densities at the electrode–solution interface.

Notes: EDC/NHS = 1-ethyl-3-(3-dimethylaminopropyl)carbodiimide/N-hydroxysuccinimide; PBSE/PyBSE = pyrene-based NHS ester linkers; CV = coefficient of variation. Reported trends refer to the nano-carbon biosensor studies charted in Section 3 and should be interpreted as route-dependent tendencies rather than universal material properties. Approximate bond or interaction energies depend on linker chemistry, surface oxidation density, pH, ionic strength, temperature, and biological matrix composition.

**Table 2:** Comparative assessment of covalent EDC/NHS and non-covalent π–π stacking biofunctionalization routes across five translational criteria (Section 4 set, *n* = 34 studies).

Criterion	Covalent EDC/NHS	Non-Covalent π–π Stacking (PBSE/PyBSE/PBASE)
sp^2^ integrity	Depends on pre-existing or oxidation-introduced –COOH groups. In graphitic materials, higher oxidation or defect density may disrupt the sp^2^ network and reduce electronic performance, particularly in FET platforms.	Better preserved because the linker adsorbs through π–π interactions without forming a covalent bond directly with the basal plane. This is advantageous for FET devices that depend on carrier mobility and field-effect sensitivity.
Bond/linker stability	High stability after amide-bond formation between surface –COOH groups and amine-containing bioreceptors. However, coupling efficiency depends strongly on –COOH density, activation conditions, and hydrolysis of activated esters.	Moderate to high under many buffer-based assay conditions but governed by non-covalent pyrene–carbon adsorption. Long-term retention and dissociation kinetics in undiluted biological matrices remain insufficiently characterized.
Bioreceptor orientation	Partial or limited. EDC/NHS can react with multiple accessible amines on antibodies or proteins, generating mixed orientations and possible antigen-binding-site occlusion.	Potentially improved at the surface level because the carbon–linker interaction is separated from bioreceptor coupling. However, NHS–amine chemistry still does not guarantee site-specific orientation unless combined with oriented capture strategies.
Lot-to-lot reproducibility	Potentially low when surface –COOH density, oxidation degree, supplier source, or post-treatment protocols are not controlled. These variables can propagate into differences in receptor loading and analytical response.	Potentially moderate, provided that sp^2^ surface quality is consistent. However, adsorption efficiency may still vary with graphene/rGO/CNT quality, contamination, defect density, and batch-to-batch nanomaterial heterogeneity.
Main translational limitation	Reproducibility. Surface heterogeneity and variable carboxyl density can produce batch-dependent coupling efficiency and LOD variability that may not be detected in buffer-only validation.	Matrix stability. The k_off of the pyrene–carbon complex, receptor retention and signal drift in undiluted clinical matrices with competing biomolecules remain insufficiently reported in the Section 4 dataset.

Notes: EDC = 1-ethyl-3-(3-dimethylaminopropyl)carbodiimide hydrochloride; NHS = N-hydroxysuccinimide; PBSE = pyrene butanoic acid succinimidyl ester; PyBSE = 1-pyrenebutanoyl succinimidyl ester; k0 = heterogeneous electron-transfer rate constant; rGO = reduced graphene oxide; sp2 = carbon hybridization state associated with planar conjugated networks. Set-of-studies frequencies in Supplementary Material S3 indicate EDC/NHS as the primary route in 16/34 studies (47%), or 18/34 (53%) if mixed EDC/NHS routes are included. Pi–pi/pyrene-mediated interactions are identifiable only in mixed or adsorptive routes (3/34, 8.8%) and not as an exclusive dominant route. Stability and kinetic values derived from literature benchmarks are cited in Section 4.2; no included study reported k_off under competitive aromatic conditions.

**Table 3:** Transduction platforms identified across the 18 Section 5 nano-carbon cancer biosensor studies: physical principle, representative nano-carbon or hybrid materials, architectural strengths, critical limitations, study count, and representative references.

Platform	Principle	NC Families	Strength	Limitation	*n*	Representative References
Voltametric (DPV/SWV)	Faradaic redox at the electrode	MWCNT, Gr/GO, CDs	Frequently reported fM–aM LODs	Inter-lot CV rarely reported; serum matrix effects incompletely characterized	3	[40,41]
Ratiometric electrochemical	Dual-signal internal reference	MWCNT, CDs	Compensates matrix-induced drift; reduces systematic error	Requires two redox reporters; increased fabrication complexity	1	[38]
Impedimetric (EIS)	ΔR_CT on binding	Gr–PBSE	Label-free; captures binding kinetics without redox mediator	Potentially narrow dynamic range *n*	1	[56]
Amperometric (I–t)	Nanozyme H_2_O_2_ catalysis	rGO@PB/Pt	In-situ metabolite readout; compatible with clinical sample volumes	Requires a constant applied potential; susceptible to O_2_ interference	1	[43]
Field-effect transistor (FET)	Channel charge modulation	CNT, GQD, Gr	Label-free; attomolar LODs; integrable with CMOS	Humidity and pH sensitivity; CNT batch-to-batch CV uncontrolled	4	[45,46,47,48]
Fluorescence	Photon emission from 0D NC	CDs, CQDs	High signal-to-noise ratio in saliva; no electrode required	Photobleaching under prolonged excitation; endogenous fluorophore interference	2	[50,56]
Electrochemiluminescence (ECL)	Electro-triggered luminescence	Eu-CDs + Ag dendrites	Background-free readout; femtomolar LODs	Co-reactant cost; laboratory-bound instrumentation	1	[51]
Wearable molecularly imprinted sensing	Binding-induced electrochemical or resistive signal change in a flexible MIP-coated interface	LIG, Gr/MIP	Antibody-free recognition; flexible format; compatible with decentralized testing	Selectivity drift, fouling, and polymer swelling under prolonged use remain insufficiently characterized	1	[37]
SPR/metasurface/THz	Plasmon shift with Δn	CNT, Gr, BP, MXene	Multi-band, label-free; high theoretical sensitivity	No experimental validation (3/3 studies computational only) †	3	[52,53,54]

Notes: † SPR/metasurface/THz platforms are included to map an emerging design space; their LODs are theoretical estimates and are not directly commensurable with experimentally measured values. Abbreviations: NC = nano-carbon; DPV = differential pulse voltammetry; SWV = square-wave voltammetry; EIS = electrochemical impedance spectroscopy; FET = field-effect transistor; ECL = electrochemiluminescence; SPR = surface plasmon resonance; THz = terahertz; Gr = graphene; GO = graphene oxide; rGO = reduced graphene oxide; MWCNT = multi-walled carbon nanotube; CDs = carbon dots; CQDs = carbon quantum dots; GQD = graphene quantum dot; BP = black phosphorus; CV = coefficient of variation; LOD = limit of detection; RCT = charge-transfer resistance.

**Table 4:** Reproducibility evidence pyramid across the Section 7 set (*n* = 87 studies).

Reporting Category	*n* Studies	% of Working Set
Do not mention batch reproducibility	65	74.7%
Mention batch behavior without quantifying CV	19	21.8%
Quantify inter-batch CV (reported)	3	3.4%
Achieve inter-batch CV ≤ 10% (FDA regulatory threshold)	3	3.4%
Report data in real clinical samples	25	28.7%
Include formal clinical comparator with agreement metric	8	9.2%

Notes: CV = coefficient of variation. Percentages were calculated using the Section 7 working set as denominator (*n* = 87). The first three reproducibility categories are mutually exclusive and sum to the full working set. The CV ≤ 10% category is a subset of studies that quantified inter-batch CV. Real-clinical-sample testing and formal comparator-based validation are separate translational evidence dimensions and are therefore not additive with the reproducibility categories. The CV ≤ 10% threshold is used here as a conservative reproducibility benchmark for the Translational Readiness Matrix.

**Table 5:** Eighty-seven biosensing platforms occupying the Real-matrix/Clinical tier of the Translational Readiness Matrix (2024–2026).

Study	Cancer Type	Target Biomarker/Signature	Biological Matrix	Transducer Architecture	Clinical Cohort Size	TRL Placement/Evidence Basis	Comparator/Agreement Metric	Reported LOD
[66]	Lung cancer	4-plex panel: NSE, CEA, p53, SOX2	Clinical specimens; matrix NR in charting	Multiplexed LIG electrochemical immunosensor	*n* = 105	Real-matrix/Clinical; cohort-confirmed with comparator/model validation	AUC = 0.936; independent validation reported	p53 = 1.62 pg/mL; CEA = 2.79 pg/mL; NSE = 3.18 pg/mL; SOX2 = 1.69 pg/mL
[92]	Lung cancer	Protease activity signature	Human blood/serum	Electrochemical activity-based enzyme biosensor	750 assays/450 unique donors	Real-matrix/Clinical; strongest comparator-anchored exemplar	Clinical cancer status; sensitivity 90%, specificity 82%, stage I sensitivity 90%	NR/not applicable
[93]	Lung cancer	EpCAM	Serum/biological fluids from metastatic lung cancer patients	Microfluidic electrochemical immunosensor using AgNPs@GO	NR	Real-matrix/Clinical; patient-matrix evaluation, comparator NR	NR	1.12 pg/mL
[94]	Lymphoma	Oncoprotein c-Myc	Serum from lymphoma patients	Dual-mode EC/ECL immunosensor using AuMrGO and Tri-Ru label	NR	Real-matrix/Clinical; patient-serum evaluation, comparator NR	NR	ECL: 4.9 pg/L; EC: 30 pg/L
[95]	Liver disease/liver cancer context	EV miRNAs: miR-21 and miR-155	Human plasma, clinical EVs	CNT-FET with tetrahedral DNA probes	NR	Real-matrix/Clinical; clinical EV matrix, comparator NR	NR	miR-21: 0.603 aM; miR-155: 1.09 aM
[96]	NSCLC	PD-L1-positive CTCs/CTC capture	Blood from NSCLC patients	Portable magnetic electrochemical sensor with N-CQDs and magnetic beads	*n* = 41 NSCLC patients	Real-matrix/Clinical; confirmed patient blood, comparator NR	NR	PD-L1: 2 ng/mL
[98]	Colorectal cancer	Circulating tumor cells	Whole blood	Electrochemical MWCNT aptasensor	*n* = 10; 5 healthy + 5 CRC	Real-matrix/Clinical; small confirmed clinical cohort, comparator NR	No formal diagnostic comparator reported	3 cells/mL
[97]	Colorectal cancer	piR-54265	Diluted human serum	Solution-gated graphene FET	NR	Real-matrix/Clinical; clinical serum matrix, comparator NR	ROC AUC = 1.0 reported; formal reference-method comparator NR	8.56 × 10^−19^ M

Notes: TRL = Technology Readiness Level; NR = not reported in the charted source; LOD = limit of detection; LIG = laser-induced graphene; AgNPs@GO = silver nanoparticles on graphene oxide; EC = electrochemical; ECL = electrochemiluminescence; AuMrGO = gold nanoparticle-modified magnetic reduced graphene oxide; CNT-FET = carbon nanotube field-effect transistor; EV = extracellular vesicle; CTC = circulating tumor cell; NSCLC = non-small cell lung cancer; CRC = colorectal cancer. All rows are classified as Real-matrix/Clinical in the Translational Readiness Matrix, but comparator-anchored validation is present only in a subset; therefore, these entries should be interpreted as upper-bound translational evidence rather than validated clinical diagnostics.

## Data Availability

All data generated or analyzed during this scoping review are included in the article and its Supplementary Materials. Supplementary Material S1 contains the PRISMA-ScR a priori protocol in standard PRISMA-ScR reporting format, including the completed PRISMA-ScR item map. Supplementary Material S2 contains the detailed methodological protocol, search strategy, methodological appendices, Translational Readiness Matrix criteria, exclusion log, PRISMA-ScR flow diagram (Figure S1), and minimum reporting checklist. Supplementary Material S3 contains the Master Charting workbook with the per-study extracted data used for the descriptive analyses, tables, figures, Evidence Level assignments, and TRL-inspired classifications.

## Supplementary Materials

The following supporting information can be downloaded at https://www.mdpi.com/article/10.3390/bios16070395/s1, Supplementary Material S1, the PRISMA-ScR a priori protocol in standard PRISMA-ScR reporting format, including the completed PRISMA-ScR item map; Supplementary Material S2, detailed methodological protocol and complete search strategy, including the PCC eligibility framework, database-specific search strings, source-selection process, data-charting plan, Translational Readiness Matrix criteria, exclusion log, PRISMA-ScR flow diagram (Figure S1), and minimum reporting checklist; Supplementary Material S3, Master Charting workbook, including the per-study extracted data used for the descriptive synthesis, with section-specific charting of nano-carbon family, surface chemistry, biorecognition strategy, transduction modality, biological matrix, analytical performance, reproducibility, stability, comparator evidence, Evidence Level, and TRL-inspired stage. References [11,13,14,15,16,17,18,113,114,115,116,117,118,119,120] are cited in the Supplementary Materials.

## Abbreviations

AFP, alpha-fetoprotein; ANVISA, Agência Nacional de Vigilância Sanitária (Brazilian Health Regulatory Agency); CA-125, cancer antigen 125; CA-15-3, cancer antigen 15-3; CD, carbon dot; CEA, carcinoembryonic antigen; CNT, carbon nanotube; CTC, circulating tumor cell; ctDNA, circulating tumor DNA; DPV, differential pulse voltammetry; ECL, electrochemiluminescence; EDC, 1-ethyl-3-(3-dimethylaminopropyl)carbodiimide hydrochloride; EIS, electrochemical impedance spectroscopy; ELISA, enzyme-linked immunosorbent assay; EUREL, 71. Convention of National Associations of Electrical Engineers in Europe; FDA, U.S. Food and Drug Administration; FET, field-effect transistor; GO, graphene oxide; GQD, graphene quantum dot; HER2, human epidermal growth factor receptor 2; HRP, horseradish peroxidase; IARC, International Agency for Research on Cancer; IME, Instituto Militar de Engenharia (Brazil); INCA, 2. Instituto Nacional de Câncer (Brazil); IVDR, In Vitro Diagnostic Medical Devices Regulation (European Union); JBI, Joanna Briggs Institute; LbL, layer-by-layer; LIG, laser-induced graphene; LOD, limit of detection; LOQ, limit of quantification; LSPR, localized surface plasmon resonance; MAGUS, magnetism-assisted graphene-based ultrasensitive sensing (reference architecture); MIP, molecularly imprinted polymer; MOF, metal–organic framework; MWCNT, multi-walled carbon nanotube; NHS, N-hydroxysuccinimide; NSE, neuron-specific enolase; NSCLC, non-small-cell lung cancer; OSCC, oral squamous-cell carcinoma; PBSE, 1-pyrenebutyric acid N-hydroxysuccinimide ester; PCC, Population, Concept, Context (eligibility framework); PD-L1, programmed death-ligand 1; PNA, peptide nucleic acid; POC, point-of-care; PRISMA-ScR, Preferred Reporting Items for Systematic Reviews and Meta-Analyses extension for Scoping Reviews; PSA, prostate-specific antigen; RCA, rolling circle amplification; rGO, reduced graphene oxide; SPCE, screen-printed carbon electrode; SPR, surface plasmon resonance; SWCNT, single-walled carbon nanotube; SWV, square-wave voltammetry; TRL, technology readiness level; TRM, Translational Readiness Matrix (instrument introduced in this review); μPAD, microfluidic paper-based analytical device.
