# Supplementary material for: Nano-Carbon Biointerfaces in Biosensors for Cancer: A Scoping Review Mapping the Transition from Proof-of-Concept to Translational Applicability (2024–2026)
Source: Biosensors (Basel). 2026 Jul 21;16(7):395. doi: 10.3390/bios16070395 (PMC13406662; doi:10.3390/bios16070395)
Supplement: Supplementary file 1 [file biosensors-16-00395-s001.zip › biosensors-4383214-Index_Supplementary_Materials.pdf]

Index of Supplementary Materials and Appendices

Manuscript biosensors-4383214 — Nano-Carbon Biointerfaces in Biosensors for Cancer: A Scoping Review

Supplementary Material S1

biosensors-4383214-Supplementary_Material_PRISMA-ScR.docx (8 pages)

A priori PRISMA-ScR protocol — numbered Sections 1–14.

Label	Name / content	Page
**Sections 1–5**	Review Type · Objective · Protocol and Registration · PCC Framework · Eligibility Criteria	p.1–2
**Section 6**	Information Sources	p.3
**Section 7**	Complete Electronic Search Strategies	p.4
**Sections 8–9**	Selection of Sources of Evidence · Data Charting Process	p.5
**Sections 10–11**	Critical Appraisal · Synthesis of Results	p.6
**Section 12**	PRISMA-ScR Item Map (22 items)	p.6
**Sections 13–14**	Funding and Role of Funders · References	p.7

Supplementary Material S2

biosensors-4383214-Supplementary_Material_S2.docx (22 pages)

Methodological protocol and appendices — cited in the main text as S2.1–S2.9.

In manuscript	In file	Name / content	Page
**S2 (body)**	—	A priori protocol (Introduction, Review questions, Inclusion criteria, Methods…)	p.1–6
**S2.1**	Appendix A	Sequential appendix map + nine-item minimum reporting checklist	p.7
**S2.2**	Appendix B	Final electronic search strings (per database)	p.9
**S2.3**	Appendix C	Translational Readiness Matrix — extraction instrument	p.10
**S2.4**	Appendix D	Complete database-specific search strategies	p.11
**S2.5**	Appendix E	Data extraction/charting instrument (42 fields)	p.13
**S2.6**	Appendix F	Translational Readiness Matrix — Evidence-Level rubric (F.1 on p.17)	p.15
**S2.7**	Appendix G	Exclusion log and final corpus update	p.19
**S2.8**	Appendix H	PRISMA-ScR flow information	p.21
**S2.9**	Appendix I	Population–Concept–Context (PCC) framework	p.22

Tables and figure in Supplementary Material S2

In manuscript	In file	Name / content	Page
**Table S1**	Table S1	Records retrieved by database	p.12 (in S2.4)
**Table S2**	Table S2	Charting fields (42 items) with operational definitions	p.13 (in S2.5)
**Table S3**	Table S3	Population–Concept–Context framework table	p.22 (in S2.9)
**Figure S1**	Figure S1	PRISMA-ScR flow diagram of the scoping review	p.21 (in S2.8)

Supplementary Material S3

biosensors-4383214-Supplementary_Material_Sections.xlsx (Excel workbook)

In manuscript	Worksheet	Content	Size
**S3**	Resumo_secoes	Summary of sections (overview)	6 rows
**S3, Section 3 charting**	Sec_3	Nano-carbon families	232 rows
**S3, Section 4 charting**	Sec_4	Surface chemistry / biointerface	36 rows
**S3, Section 5 charting**	Sec_5	Transduction platforms	20 rows
**S3, Section 6 charting**	Sec_6	Targets/biomarkers and matrices	76 rows
**S3, Section 7 charting**	Sec_7	Translational readiness	129 rows
