# Supplementary material for: Nano-Carbon Biointerfaces in Biosensors for Cancer: A Scoping Review Mapping the Transition from Proof-of-Concept to Translational Applicability (2024–2026)
Source: Biosensors (Basel). 2026 Jul 21;16(7):395. doi: 10.3390/bios16070395 (PMC13406662; doi:10.3390/bios16070395)
Supplement: Supplementary file 1 [file biosensors-16-00395-s001.zip › biosensors-4383214-Supplementary_Material_PRISMA-ScR_S1.pdf]

Supplementary Material S1

PRISMA-ScR a priori protocol and complete search strategy

This supplementary material reports on the methodological protocol, eligibility framework, complete electronic search strategies, selection process, data-charting plan, and PRISMA-ScR item map used for the scoping review. It is intentionally limited to methods and reporting support; per-study extracted data are provided separately in Supplementary Material 2.

ABSTRACT

Cancer remains a leading global health burden, and nano-carbon biointerfaces have emerged as a structurally versatile platform for sensitive, biorecognition-enabled detection of cancer biomarkers. The breadth of nano-carbon families, surface chemistries, transduction principles, and biological matrices in recent literature has outpaced systematic synthesis, motivating a mapping exercise of methodological and translational status. Methods: This scoping review follows the JBI 2024 methodology and the PRISMA-ScR 2018 reporting framework, using the Population–Concept–Context (PCC) eligibility scheme. Six bibliographic databases (PubMed, Scopus, Web of Science, IEEE Xplore, ScienceDirect, LILACS) were searched on 18 May 2026, with corpus closure on 31 May 2026; 1,689 records were identified, and 191 primary studies were charted (a priori protocol and PRISMA-ScR item map in Supplementary Material 1; per-study charting in Supplementary Material 2). No critical appraisal of individual sources was performed, in line with the scoping review methodology. Results: Three research questions were addressed. RQ1 maps the design space (nano-carbon family × surface chemistry × transduction modality) across zero-dimensional, one-dimensional, two-dimensional, and three-dimensional hybrid nano-carbon platforms; reported LODs do not monotonically track dimensionality. RQ2 maps the analytical performance envelope against the biological matrix, revealing matrix-conditioned modality choice. RQ3 maps translational maturity: approximately 67% of studies remain at the Low Evidence Level, approximately 24% at the Intermediate Evidence Level, and approximately 10% at the Strong Evidence Level, while the Clinical TRL bracket remains sparse. Conclusions: Nano-carbon biointerfaces are technically mature for cancer biomarker detection but translationally constrained by recurring bottlenecks in reproducibility reporting, real-matrix validation, comparator agreement, regulatory pathway, manufacturing scale, workflow integration, and clinical-cohort size. The charted evidence map and gap inventory orient subsequent systematic reviews and translational research.

1. Review Type and Reporting Framework

The review was designed as a scoping review because the recent literature on nano-carbon biosensors for cancer is heterogeneous in nano-carbon family, surface chemistry, biorecognition element, transduction mode, biological matrix, and translational endpoint. This heterogeneity made formal effect-size pooling or meta-analysis inappropriate. Reporting follows the PRISMA extension for scoping reviews (PRISMA-ScR, 2018) and the JBI scoping-review approach, using the Population–Concept–Context (PCC) framework.

No formal critical appraisal or risk-of-bias assessment of individual sources was performed. The Translational Readiness Matrix used in the manuscript is a descriptive mapping framework and should not be interpreted as a measure of methodological quality.

2. Objective and Research Questions

Objective: To map how nano-carbon biointerfaces were designed and applied as cancer biosensors in the 2024–2026 literature, and to identify material, analytical, and translational determinants relevant to the transition from proof-of-concept devices toward clinically usable evidence.

- RQ1: What nano-carbon families, surface chemistries, biorecognition elements, and transduction modalities are being used in cancer biosensors?
- RQ2: How are analytical performance and biological-matrix validation reported across the mapped studies?
- RQ3: What level of translational maturity is indicated by the reported validation context, sample type, comparator use, and clinical proximity?

3. Protocol and Registration

An a priori protocol was prepared to guide the scope, eligibility criteria, search strategy, screening, data charting, and descriptive synthesis. A prospective public registry record was not documented in the materials provided; therefore, this review should be described as protocol-guided but not prospectively registered. The final electronic searches were run on 18 May 2026, and the corpus was closed on 31 May 2026, consistent with the 2024–2026 eligibility window.

4. PCC Framework

PCC element	Operational definition used in the review
Population	Adults and adolescents with confirmed or suspected solid tumor or hematological cancer for whom biomarker measurement was reported. Preclinical animal studies were excluded from the primary corpus but could be retained as contextual references.
Concept	Nano-carbon biointerfaces: biosensor architectures incorporating one or more nano-carbon families, including carbon dots, graphene/GO/rGO, carbon nanotubes, fullerenes, nanodiamonds, MXene-carbon hybrids, or three-dimensional composites, coupled to a biorecognition element such as an antibody, aptamer, peptide, nucleic acid probe, or molecularly imprinted polymer for cancer biomarker detection.
Context	Clinical, point-of-care, wearable, liquid-biopsy, and related biosensing contexts targeting tumor biomarkers, proteins, microRNAs, circulating tumor cells/DNA, exosomes, volatile organic compounds, or other cancer-relevant analytes in 2024–2026 publications.

5. Eligibility Criteria

Domain	Include	Exclude
Publication type	Primary research articles and conference papers reporting original biosensor development, characterization, or validation.	Reviews, systematic reviews, meta-analyses, editorials, letters without original experimental data, patents, and purely theoretical work.
Time window	Publications indexed or published within the 2024–2026 review window.	Records outside the review window.
Language	English, Portuguese, or Spanish.	Languages outside the prespecified set when sufficient information could not be extracted.

Cancer relevance	Studies explicitly targeting cancer diagnosis, screening, monitoring, prognosis, or cancer biomarker detection.	Non-cancer applications or generic biomarker studies without cancer relevance.
Nano-carbon requirement	Studies using a nano-carbon material as part of the biosensor interface or signal-amplifying architecture.	Biosensors without a nano-carbon component or studies where carbon was only a bulk electrode/support without nano-scale biointerface relevance.
Biosensing requirement	Studies coupling a recognition strategy to a measurable analytical signal.	Material synthesis studies without biosensing, imaging-only studies without a biosensor readout, or therapeutic delivery studies without analytical detection.
Sample/matrix	Buffer, spiked samples, cell lysate, serum, plasma, saliva, urine, breath condensate, tissue-derived samples, exosomes, ctDNA/miRNA preparations, or other cancer-relevant matrices.	Animal-only preclinical studies from the primary corpus, unless retained only as contextual references.

6. Information Sources

The manuscript reports searches in six bibliographic sources. Database-specific search syntax was adapted to each platform while preserving the three conceptual blocks: cancer, biosensing, and nano-carbon materials.

Source	Role in search	Date/limits reported
PubMed/MEDLINE	Biomedical indexing and MeSH-based retrieval.	Search date: 18 May 2026; corpus closure: 31 May 2026.
Scopus	Broad multidisciplinary citation database.	2024–2026; English, Portuguese, Spanish.
Web of Science	Multidisciplinary citation database.	2024–2026; articles/proceedings.
IEEE Xplore	Engineering, electronics, and sensor-platform literature.	2024–2026; journals/conferences.
ScienceDirect	Publisher database searched using segmented query blocks due to platform constraints.	2024–2026; final eligibility applied during screening.
LILACS/DeCS BVS	Regional biomedical database with Portuguese/Spanish terminology.	2024–2026; DeCS/free-text terms.

7. Complete Electronic Search Strategies

The following strategies reproduce the search strings documented in the protocol. Platform syntax may require minor interface-level adaptations when rerunning the search, but conceptual blocks and eligibility limits should remain unchanged.

PubMed/MEDLINE

((("Neoplasms"[Mesh] OR "Biomarkers, Tumor"[Mesh] OR "Early Detection of Cancer"[Mesh] OR cancer*[tiab] OR neoplasm*[tiab])) AND (("Biosensing Techniques"[Mesh] OR biosensor*[tiab] OR immunosensor*[tiab] OR aptasensor*[tiab] OR genosensor*[tiab])) AND (("Nanotubes, Carbon"[Mesh] OR "Graphene"[Mesh] OR "Quantum Dots"[Mesh] OR nanocarbon*[tiab] OR graphene[tiab] OR "graphene oxide"[tiab] OR "reduced graphene oxide"[tiab] OR "carbon nanotube*"[tiab] OR SWCNT[tiab] OR MWCNT[tiab] OR "carbon dot*"[tiab] OR "graphene quantum dot*"[tiab] OR GQDs[tiab] OR nanodiamond*[tiab] OR fullerene*[tiab])) AND ("2024"[PDAT]: "2026"[PDAT]) AND (English[lang] OR Portuguese[lang] OR Spanish[lang]) NOT (Review[Publication Type] OR Systematic Review[Filter] OR Meta-Analysis[Publication Type])

Scopus

(TITLE-ABS-KEY(cancer* OR neoplasm*)) AND (TITLE-ABS-KEY(biosensor* OR immunosensor* OR aptasensor* OR genosensor*)) AND (TITLE-ABS-KEY(nanocarbon* OR graphene OR "graphene oxide" OR "reduced graphene oxide" OR "carbon nanotube*" OR SWCNT OR MWCNT OR "carbon dot*" OR "graphene quantum dot*" OR GQDs OR nanodiamond* OR fullerene*)) AND PUBYEAR > 2023 AND PUBYEAR < 2027 AND (LIMIT-TO(LANGUAGE,"English") OR LIMIT-TO(LANGUAGE,"Portuguese") OR LIMIT-TO(LANGUAGE,"Spanish")) AND (LIMIT-TO(DOCTYPE,"ar") OR LIMIT-TO(DOCTYPE,"cp"))

Web of Science

(TS=(cancer* OR neoplasm*)) AND (TS=(biosensor* OR immunosensor* OR aptasensor* OR genosensor*)) AND (TS=(nanocarbon* OR graphene OR "graphene oxide" OR "reduced graphene oxide" OR "carbon nanotube*" OR SWCNT OR MWCNT OR "carbon dot*" OR "graphene quantum dot*" OR GQDs OR nanodiamond* OR fullerene*)) AND PY=(2024–2026) AND LA=(English OR Portuguese OR Spanish) AND DT=(Article OR "Proceedings Paper")

IEEE Xplore

((cancer OR neoplasm OR tumor OR oncology)) AND ((biosensor OR immunosensor OR aptasensor OR biosensing)) AND ((nanocarbon OR graphene OR "graphene oxide" OR "reduced graphene oxide" OR "carbon nanotube" OR SWCNT OR MWCNT OR "carbon dot" OR "graphene quantum dot" OR nanodiamond OR fullerene)) AND ("Publication_Year":2024–2026) AND (Document_Type:"Journals" OR Document_Type:"Conferences")

ScienceDirect

Query A: (cancer OR tumor OR neoplasm) AND (biosensor OR immunosensor) AND (graphene OR "graphene oxide" OR "carbon nanotube")
Query B: (cancer OR tumor) AND (biosensor OR biosensing) AND ("carbon dot" OR "graphene quantum dot" OR nanocarbon)
Query C: (cancer OR tumor OR "tumor biomarker") AND (biosensor OR aptasensor) AND ("real sample" OR "clinical sample" OR plasma OR serum)
Note: Query C functioned as a real-matrix sensitivity query because of platform constraints; candidate records still had to satisfy nano-carbon eligibility during screening.

LILACS/DeCS BVS

((mh:("Neoplasias") OR mh:("Biomarcadores Tumorais") OR mh:("Deteccao Precoce de Cancer") OR tw:(cancer*) OR tw:(neoplas*))) AND ((mh:("Tecnicas Biossensoriais") OR tw:(biosensor*) OR tw:(biossensor*) OR tw:(immunosensor*) OR tw:(imunossensor*) OR tw:(aptasensor*))) AND ((mh:("Nanotubos de Carbono") OR mh:("Grafite") OR tw:(nanocarbon*) OR tw:("nanocarbono") OR tw:(grafeno) OR tw:(graphene) OR tw:("oxido de grafeno") OR tw:("graphene oxide") OR tw:("nanotubo* de carbono") OR tw:("carbon nanotube*") OR tw:("ponto* de carbono") OR tw:("carbon dot*") OR tw:("graphene quantum dot*") OR tw:(GQDs) OR tw:(nanodiamante) OR tw:(nanodiamond) OR tw:(fulereno) OR tw:(fullerene))) AND year_cluster:[2024 TO 2026]

8. Selection of Sources of Evidence

Records retrieved from all sources were de-duplicated and screened in stages. Titles and abstracts were screened first against the PCC framework and eligibility criteria. Potentially relevant records then underwent full-text assessment.

Disagreements or uncertain classifications were resolved by discussion using the predefined screening rubric. Rayyan was used to support screening; software version and reviewer allocation were not retained in the final audit trail and are therefore not treated as analytic variables.

PRISMA-ScR flow stage	Number/status reported	Comment for final submission
Identification	1689 records identified.	Reported in the manuscript abstract.
Screening after de-duplication	975 records entered the PRISMA-ScR flow/screening set.	Reported in the manuscript methods; use as post-deduplication screening denominator.
Eligibility	Full-text eligibility assessed before final charting.	Full-text assessment was performed before inclusion; a separate full-text-assessed denominator was not reconstructable from the final audit materials.
Included/charted	191 primary studies charted.	Reported consistently in the manuscript abstract and methods.

9. Data Charting Process

Data charting was performed with a structured extraction form aligned with the PCC framework and the three research questions. Extracted fields were used for descriptive mapping rather than effect-size synthesis. When a field was not reported in a source, it was marked as not reported rather than inferred.

Domain	Data items charted
Bibliographic information	Authors, year, title, journal/conference, DOI, country/region where reported, and language.
Cancer and biomarker target	Cancer type, intended use, analyte/biomarker, biomarker class, and clinical or analytical rationale where reported.
Nano-carbon biointerface	Nano-carbon family, composite/hybrid components, synthesis or fabrication route, surface functionalization, biorecognition element, and blocking/antifouling strategy.
Biosensing platform	Transduction modality, electrode/substrate or device format, assay workflow, signal generation/amplification strategy, and measurement conditions.
Analytical performance	Limit of detection, linear/dynamic range, assay time, selectivity/interference, reproducibility, stability, recovery, and comparator/reference method where reported.
Validation matrix	Sample type, spiked or real biological matrix, real-sample number when reported, clinical sample type, and validation context.
Translation mapping	Translational Readiness Matrix category, strongest reported endpoint, clinical proximity, and limitations relevant to implementation.

10. Critical Appraisal

Consistent with the manuscript, no formal critical appraisal, risk-of-bias assessment, certainty grading, or methodological quality scoring of individual sources was performed. This choice is coherent with a scoping review whose purpose

is to map the extent, range, characteristics, and translational maturity of the evidence rather than to estimate pooled intervention effects or diagnostic accuracy parameters.

11. Synthesis of Results

Results were synthesized descriptively and narratively. Mapping focused on nano-carbon dimensionality and family, biointerface design, recognition chemistry, transduction modality, analytical-performance reporting, validation matrix, and translational-readiness category. No meta-analysis, pooled sensitivity/specificity calculation, or formal quantitative certainty assessment was planned or performed.

Translational Readiness Matrix level	Operational meaning
Low/proof-of-concept or analytical	Performance demonstrated mainly in buffer, simplified media, or early analytical conditions, with limited biological-matrix validation.
Intermediate/analytical or real-matrix validation	Performance was extended to spiked or real biological matrices, with evidence of selectivity, recovery, or matrix effect, but with limited direct clinical validation.
Strong/clinical-proximity validation	Evidence includes confirmed clinical specimens, comparison with a reference approach, or validation conditions closer to intended use; this is a descriptive translational mapping category, not a diagnostic quality score.

12. PRISMA-ScR Item Map

The table below indicates where each PRISMA-ScR reporting item is addressed across the manuscript and supplementary files. Items involving critical appraisal are marked as not applicable because individual-source appraisal was not conducted.

PRISMA-ScR item	Where addressed
1 Title	The main manuscript title identifies the report as a scoping review.
2 Structured summary	Main manuscript abstract.
3 Rationale	Main introduction and Supplementary Material 1, Section 1.
4 Objectives	Main manuscript and Supplementary Material 1, Section 2.
5 Protocol and registration	Supplementary Material 1, Section 3.
6 Eligibility criteria	Supplementary Material 1, Sections 4-5.
7 Information sources	Supplementary Material 1, Section 6.
8 Search	Supplementary Material 1, Section 7.
9 Selection of sources of evidence	Supplementary Material 1, Section 8.
10 Data charting process	Supplementary Material 1, Section 9.
11 Data items	Supplementary Material 1, Section 9; per-study charting in Supplementary Material 2.

12 Critical appraisal of individual sources	Not applicable; no formal critical appraisal was performed.
13 Synthesis of results	Supplementary Material 1, Section 11 and main manuscript methods.
14 Selection of sources of evidence	Main manuscript methods/results and Supplementary Material 1, Section 8.
15 Characteristics of sources of evidence	Main manuscript results and Supplementary Material 2.
16 Critical appraisal within sources	Not applicable.
17 Results of individual sources	Supplementary Material 2.
18 Synthesis of results	Main manuscript results and discussion.
19 Summary of evidence	Main manuscript discussion.
20 Limitations	Main manuscript discussion.
21 Conclusions	Main manuscript conclusion.
22 Funding	Main manuscript funding/conflict-of-interest statements; Supplementary Material 1, Section 13.

13. Funding and Role of Funders

Funding, author contribution, and conflict-of-interest statements should match the final main manuscript. No funder role in eligibility determination, screening, data charting, or synthesis was documented in the protocol materials available for this supplementary file.

14. References

1. Tricco, A. C.; Lillie, E.; Zarin, W.; O'Brien, K. K.; Colquhoun, H.; Levac, D.; Moher, D.; Peters, M. D. J.; Horsley, T.; Weeks, L.; et al. PRISMA Extension for Scoping Reviews (PRISMA-ScR): Checklist and Explanation. *Ann. Intern. Med.* **2018**, *169*, 467–473. https://doi.org/10.7326/M18-0850.
2. Peters, M.D.J.; Marnie, C.; Tricco, A.C.; Pollock, D.; Munn, Z.; Alexander, L.; McInerney, P.; Godfrey, C.M.; Khalil, H. Updated methodological guidance for the conduct of scoping reviews. *JBI Evid. Synth.* **2020**, *18*, 2119–2126.
3. Pollock, D.; Peters, M.D.; Khalil, H.; McInerney, P.; Alexander, L.; Tricco, A.C.; Evans, C.; de Moraes, E.B.; Godfrey, C.M.; Pieper, D.; et al. Recommendations for the extraction, analysis, and presentation of results in scoping reviews. *JBI Evid. Synth.* **2022**, *21*, 520–532.
4. PRISMA Statement. PRISMA-Scoping Reviews. https://www.prisma-statement.org/scoping (accessed on 18 May 2026).
