# Supplementary material for: Nano-Carbon Biointerfaces in Biosensors for Cancer: A Scoping Review Mapping the Transition from Proof-of-Concept to Translational Applicability (2024–2026)
Source: Biosensors (Basel). 2026 Jul 21;16(7):395. doi: 10.3390/bios16070395 (PMC13406662; doi:10.3390/bios16070395)
Supplement: Supplementary file 1 [file biosensors-16-00395-s001.zip › biosensors-4383214-Supplementary_Material_S2.pdf]

Supplementary Material S2

PRISMA-ScR a priori protocol and complete search strategy

This supplementary material reports on the methodological protocol, eligibility framework, complete electronic search strategies, selection process, data-charting plan, and PRISMA-ScR item maps used for the scoping review. It is intentionally limited to methods and reporting support; per-study extracted data are provided separately in Supplementary Material S3.

ABSTRACT

Abstract: Nano-carbon biointerfaces offer versatile platforms for cancer biomarker detection, but their progression from analytical proof-of-concept to clinically usable diagnostic evidence remains uneven. This scoping review maps 191 primary studies published from 2024 to 2026, covering nano-carbon families, surface chemistries, transduction architectures, biological matrices, and translational endpoints in cancer biosensing. The evidence space spans four nano-carbon dimensional classes: zero-dimensional carbon dots and quantum dots; one-dimensional carbon nanotubes; two-dimensional graphene-derived materials; and three-dimensional hybrid composites. Across these platforms, analytical sensitivity did not scale monotonically with nano-carbon dimensionality; instead, performance was shaped by interactions among material architecture, biointerface chemistry, recognition strategy, transduction modality, and matrix context. A Translational Readiness Matrix showed that approximately 67% of studies remained at Low Evidence Level, whereas only approximately 10% reached Strong Evidence Level. Five recurring bottlenecks constrained translation: incomplete reporting of reproducibility, limited Real-matrix Validation, scarce comparator-based clinical evidence, insufficient manufacturing-scale data, and weak regulatory or deployment planning. To address these gaps, this review proposes a nine-item minimum reporting checklist and a four-stage validation roadmap to support more reproducible, comparable, and clinically oriented nano-carbon biosensor development.

Keywords: nano-carbon biosensors; cancer diagnostics; scoping review; translational readiness; biointerfaces; limit of detection; clinical translation; regulatory pathway; nanomaterials

Introduction

Cancer is responsible for approximately 10 million deaths annually worldwide (GLOBOCAN/IARC) and over 230,000 deaths per year in Brazil (INCA). Five-year survival is strongly stage-dependent, and timely access to decentralized diagnostic testing remains a bottleneck, particularly in low- and middle-income regions. This context sustains the growing interest in point-of-care (POC) analytical platforms capable of detecting tumor biomarkers with clinically relevant sensitivity, low cost, and short turnaround time.

Biosensors couple a biological recognition element (antibodies, aptamers, peptides, enzymes, nucleic acid probes) to a transducer (electrochemical, optical, FET, mechanical, magnetic-assisted). The choice of the interface between bio-recognition and transduction, the "biointerface", determines selectivity, stability, and reproducibility. Nano-carbon materials, graphene and graphene oxide (GO/rGO), carbon nanotubes (SWCNT/MWCNT), carbon dots, graphene quantum dots (GQDs), nanodiamonds, fullerenes, and magnetic hybrids (Fe_3O_4/GO, Fe_3O_4/rGO), have been intensively investigated as biointerfaces due to their high surface area, tunable conductivity, versatile functionalization chemistry, and microfabrication compatibility.

The literature is dominated by individual proof-of-concept reports with methodological heterogeneity that hampers horizontal comparison and critical interpretation of each platform's actual technological maturity. Narrative reviews

on nano-carbon biosensors are frequent but rarely accompanied by a critical map distinguishing analytical performance (LOD, linear range, selectivity in buffer) from translational readiness (real-matrix validation, batch-to-batch reproducibility, prolonged stability, regulatory compatibility).

A preliminary search of MEDLINE, the Cochrane Database of Systematic Reviews, and JBI Evidence Synthesis was conducted on 7 May 2026, and no current or in-progress scoping review was identified that simultaneously (i) applies the PRISMA-ScR framework, (ii) covers the five main nano-carbon families, (iii) systematically classifies evidence by translational readiness levels, and (iv) explicitly addresses the trade-off between sensitivity and biomedical applicability for cancer detection in the most recent biennium.

A scoping review [1] is the most appropriate design for this question because it combines high heterogeneity across platforms, interventions, and analytical endpoints with an exploratory, non-comparative focus. These are the conditions for which [2], refined by [3] and updated by [4] and [5][6], recommend scoping over systematic reviews [7]. The JBI methodology is adopted as the operational framework, incorporating both the original conceptual basis and subsequent refinements [2]. The objective of this scoping review is to map how nano-carbon materials are being designed and applied as biointerfaces in biosensors for cancer biomarker detection in the 2024–2026 biennium, identifying the material, functional, analytical, and translational factors that determine their biomedical relevance, and building a validation agenda anchored in the available evidence.

Review questions

Main question:

How are nano-carbon-based materials being designed and applied as biointerfaces in biosensors for cancer in 2024–2026, and which material, functional, analytical, and translational factors determine their biomedical relevance?

Sub-questions:

- Which nano-carbon families dominate the recent literature, and in which oncological applications?
- Which biological matrices have been effectively validated, and in which is evidence still restricted to spiked samples?
- What is the evidence on batch-to-batch reproducibility, prolonged stability, and quantified matrix effects?
- Where are the most critical gaps for clinical translation of nano-carbon biosensors?
- What is the current and potential role of magnetic-assisted architectures (MAGUS-like, Fe_3O_4/graphene, Fe_3O_4/rGO) as an integrating element of the biointerface?

Inclusion criteria

Participants

Studies investigating biosensors (or biosensing platforms) applied to biomedical analytes with emphasis on cancer and tumor biomarkers. Eligible analytes include tumor proteins (CA 15-3, CEA, PSA, HER2, AFP, others), nucleic acids (miRNA, ctDNA, others), exosomes/extracellular vesicles, circulating tumor cells (CTCs), and micro-organisms with oncological association (H. pylori, HPV, others).

Concept

Biointerfaces based on nano-carbon materials encompass surface chemistry, biofunctionalization strategies, transduction principles, and analytical validation of molecular recognition. Eligible families include 0D (carbon dots, GQDs,

nanodiamonds, fullerenes), 1D (SWCNT, MWCNT, carbon nanofibers), 2D (graphene, graphene oxide, reduced graphene oxide), and 3D-hybrids (including magnetic-assisted architectures Fe_3O_4/GO and Fe_3O_4/rGO).

Context

Eligible contexts include the full continuum from development and analytical validation to Real-matrix Validation, point-of-care, wearable devices, lab-on-a-chip integration, and liquid biopsy. Theragnostic extensions may be included, provided an explicit biosensing component is described; purely therapeutic studies are excluded.

Types of sources

This scoping review will include primary research studies (original journal articles, conference proceedings indexed in Scopus/Web of Science/IEEE Xplore, and indexed book chapters) with extractable quantitative analytical data (LOD, linear range, selectivity).
Excluded: narrative and systematic reviews, meta-analyses, prior scoping reviews (cited as contextual references but not extracted); editorials, letters, errata, retractions, perspectives < 2 pages; patents; non-indexed theses/dissertations; publications in predatory journals; studies whose sole use of nano-carbon is as a therapeutic vehicle without biosensing.

Time and language criteria

Time window: 01 January 2024 to 18 May 2026 (last two complete years), reflecting the rapid evolution of nano-carbon biosensing platforms in diagnostic and point-of-care applications. Publications prior to 2024 will be included only as seminal foundational references (e.g., pioneering work on surface chemistry in graphene/GO, carbon magnetism, and the original definition of MAGUS-like architectures), with case-by-case justification in the data extraction file.
Languages: all languages.

Methods

This scoping review will be conducted in accordance with the JBI methodology for scoping reviews [2], with protocol development guided by [4] and data extraction/analysis/presentation guided by[5]. Reporting follows the PRISMA-ScR extension [8], with the flow diagram in the PRISMA 2020 format [9].

Search strategy

The search strategy follows the three-step approach recommended by JBI: (1) limited pilot search in PubMed and Scopus to identify key index terms and abstract words; (2) full search across all selected databases adapting the identified terms to each database's syntax; (3) hand-search of reference lists of all included studies (snowballing) and recent table-of-contents of core journals (*Biosensors MDPI, Biosensors and Bioelectronics, Analytical Chemistry, ACS Sensors*).
Databases searched (14–18 May 2026):

- PubMed/MEDLINE (National Library of Medicine)
- Scopus (Elsevier)
- Web of Science Core Collection (Clarivate)
- IEEE Xplore
- ScienceDirect (Elsevier; segmented strategy due to 8-term limit)
- DeCS/BVS (LILACS, Latin American coverage)

Search structure: P AND C_1 (biosensor) AND C_2 (nano-carbon) NOT (reviews). The complete search strings for each database are provided in S2.2. The full database export, PRISMA-ScR flow diagram, and screening decisions will be archived with the published review.

Source of evidence selection

Following the search, all identified citations will be collated and uploaded into Rayyan QCRI [10] under the project "Nano-Carbon Biointerfaces, Special Issue Biosensors MDPI", and duplicates will be removed. Title and abstract screening will be conducted using a three-round automated pre-classification framework simulating dual-blinded reviewer agreement:

- Round 1 (R1): composite relevance score (0–100) based on PCC term presence.
- Round 2 (R2): protocol-criteria check (5 hard exclusion criteria + 4 cumulative inclusion criteria).
- Round 3 (R3): analytical quality filter (5 cumulative criteria on LOD/linear range, selectivity, biological matrix, reproducibility, oncological target specificity).

Final decision per study follows a 2-of-3 majority rule across R1, R2, and R3, applied uniformly to all manuscript sections. All conflict and doubt cases are resolved through manual review and consensus between two human reviewers (N. Barbosa, B. Geraldino), with a third reviewer (E. Migon) as a tiebreaker. Full-text screening is then distributed by manuscript section to the responsible co-author. Reasons for exclusion at the full-text stage will be recorded and reported in the PRISMA-ScR flow diagram.

Data extraction

Data were extracted by the authors of each section using a standardized data collection instrument (provided as Supplementary Material S3), tested on 10 representative studies prior to large-scale extraction [5]. Each study is extracted by one reviewer; ≥ 20% of studies are independently extracted by a second reviewer to assess agreement. The data collection process is iterative; the model can be refined during extraction by reprocessing already extracted studies, with all changes documented in a change log.
Data items to be extracted:

- Bibliographic: authors, year, country of corresponding author, journal, document type, DOI, language.
- P, Population: analyte name, analyte class (protein/nucleic acid/exosome/CTC/micro-organism), cancer type, declared purpose (diagnosis/monitoring/screening/prognosis).
- C, Concept: nano-carbon family (0D/1D/2D/3D-hybrid), detailed composition, synthesis route, functionalization strategy (covalent/non-covalent), bioreceptor.
- Co, Context: transduction platform, device format, biological matrix tested.
- Analytical performance: LOD, LOQ, linear range, selectivity (interferents tested), device-to-device reproducibility, batch-to-batch reproducibility, stability (days), formal comparator method.
- Translational readiness: Evidence Level (Low/Intermediate/Strong) and TRL-inspired stage (PoC/Analytical/Real-matrix/Pre-clinical/Clinical), per the matrix defined in the project.
- Citable quotes; target manuscript section; reviewer notes; pre-2024 exception justification (when applicable).

Data analysis and presentation

Synthesis follows [5]: structured charting using predefined and iteratively refined categories; predominantly descriptive and narrative analysis supported by tabular maps and figures; and a presentation that makes the breadth and gaps of the literature visually accessible.
Presentation elements:

- Narrative synthesis structured by manuscript thematic sections (Sec_3 to Sec_7).
- Cross-tabular maps (nano-carbon family × analyte; transduction platform × biological matrix).
- Distribution chart of studies by translational readiness level and TRL-inspired stage.
- Logical biointerface chain (Figure 1): material → functionalization → transduction → matrix → device → biomedical use.
- Coverage heatmap: families × TRL-inspired stages, visual identification of gaps.
- Integrated discussion of translational gaps and proposed validation agenda.
- PRISMA-ScR flow diagram with real counts at each stage.

Following the guidelines of the JBI [1] and PRISMA-ScR [6], scoping reviews do not require a formal critical appraisal of individual sources of evidence. Instead of assessing risk of bias, this review, whenever possible, uses a Translational Readiness Matrix as a descriptive instrument, classifying each study into three cumulative levels of evidence and five stages, inspired by the concept of Translational Readiness Level (TRL). This classification does not represent a judgment of quality but rather a relative positioning along the bench-to-clinic axis.

Protocol registration and availability

This protocol was developed a priori following [4] and is provided in full as Supplementary Material with the corresponding manuscript submitted to the Special Issue "Nano-Carbons in Biosensors" of the journal *Biosensors (MDPI)*. The PRISMA-ScR checklist and the raw screening dataset will also be released as Supplementary Material under CC-BY 4.0 license. Any deviations from this protocol after the start of full-text screening are documented in a versioned changelog and reported transparently in the Methods section of the final manuscript.

References

1. Munn, Z.; Pollock, D.; Khalil, H.; Alexander, L.; Mclnerney, P.; Godfrey, C.M.; Peters, M.; Tricco, A.C. What are scoping reviews? Providing a formal definition of scoping reviews as a type of evidence synthesis. *JBI Evid. Synth.* **2022**, *20*, 950–952. https://doi.org/10.11124/jbies-21-00483.
2. Arksey, H.; O'Malley, L. Scoping studies: Towards a methodological framework. *Int. J. Soc. Res. Methodol.* **2005**, *8*, 19–32. https://doi.org/10.1080/1364557032000119616.
3. Levac, D.; Colquhoun, H.; O'Brien, K.K. Scoping studies: Advancing the methodology. *Implement. Sci.* **2010**, *5*, 69. https://doi.org/10.1186/1748-5908-5-69.
4. Peters, M.D.J.; Marnie, C.; Tricco, A.C.; Pollock, D.; Munn, Z.; Alexander, L.; McInerney, P.; Godfrey, C.M.; Khalil, H. Updated methodological guidance for the conduct of scoping reviews. *JBI Evid. Synth.* **2020**, *18*, 2119–2126. https://doi.org/10.11124/JBIES-20-00167.
5. Pollock, D.; Peters, M.D.J.; Khalil, H.; McInerney, P.; Alexander, L.; Tricco, A.C.; Evans, C.; de Moraes, E.B.; Godfrey, C.M.; Pieper, D.; et al. Recommendations for the extraction, analysis, and presentation of results in scoping reviews. *JBI Evid. Synth.* **2023**, *21*, 520–532. https://doi.org/10.11124/JBIES-22-00123.
6. Pollock, D.; Davies, E.L.; Peters, M.D.J.; Tricco, A.C.; Alexander, L.; McInerney, P.; Godfrey, C.M.; Khalil, H.; Munn, Z. Undertaking a scoping review: A practical guide for nursing and midwifery students, clinicians, researchers, and academics. *J. Adv. Nurs.* **2021**, *77*, 2102–2113. https://doi.org/10.1111/jan.14743.

7. Munn, Z.; Peters, M.D.J.; Stern, C.; Tufanaru, C.; McArthur, A.; Aromataris, E. Systematic Review or Scoping Review? Guidance for Authors When Choosing between a Systematic or Scoping Review Approach. *BMC Med. Res. Methodol.* **2018**, *18*, 143. https://doi.org/10.1186/s12874-018-0611-x.
8. Tricco, A.C.; Lillie, E.; Zarin, W.; O'Brien, K.K.; Colquhoun, H.; Levac, D.; Moher, D.; Peters, M.D.J.; Horsley, T.; Weeks, L.; et al. PRISMA Extension for Scoping Reviews (PRISMA-ScR): Checklist and Explanation. *Ann. Intern. Med.* **2018**, *169*, 467–473. https://doi.org/10.7326/M18-0850.
9. Page, M.J.; McKenzie, J.E.; Bossuyt, P.M.; Boutron, I.; Hoffmann, T.C.; Mulrow, C.D.; Shamseer, L.; Tetzlaff, J.M.; Akl, E.A.; Brennan, S.E.; et al. The PRISMA 2020 statement: An updated guideline for reporting systematic reviews. *BMJ* **2021**, *372*, n71. https://doi.org/10.1136/bmj.n71.
10. Ouzzani, M.; Hammady, H.; Fedorowicz, Z.; Elmagarmid, A. Rayyan, a web and mobile app for systematic reviews. *Syst. Rev.* **2016**, *5*, 210. https://doi.org/10.1186/s13643-016-0384-4.
11. Rethlefsen, M.L.; Kirtley, S.; Waffenschmidt, S.; Ayala, A.P.; Moher, D.; Page, M.J.; Koffel, J.B.; PRISMA-S Group. PRISMA-S: An extension to the PRISMA Statement for Reporting Literature Searches in Systematic Reviews. *Syst. Rev.* **2021**, *10*, 1–19, https://doi.org/10.1186/s13643-020-01542-z.

S2.1

This Supplementary Material S2 constitutes the full a priori protocol cited in Section 2 of the main manuscript. The research question and Population–Concept–Context (PCC) framing are presented in Section 2 and S2.9 (Table S3); the eligibility criteria are presented in Section 3; and the search strategy, with full database strings, is presented in Section 4 and Appendices B and D.

Sequential appendix map

This section lists the sequential appendix labels used in this Supplementary Material and indicates how they correspond to the appendix labels cited in the manuscript.
Based on the reporting gaps identified across Sections 4, 5, 7, and 8, we propose the following checklist for future nano-carbon biosensor studies. The checklist is intended to improve reproducibility, comparability, and translational interpretability. Items 1–6 apply broadly to nano-carbon biosensor publications, whereas Items 7–9 are context-dependent and should be reported when studies make claims related to patient contact, point-of-care use, wearable deployment, connected diagnostics, lab-on-chip integration, or regulatory translation.

1. Nano-carbon material and precursor traceability. Studies should report the nano-carbon family, precursor source, supplier, and batch number when the material is commercially obtained. For graphene-based and other two-dimensional materials, reporting should include the C/O ratio from XPS, the Raman D/G ratio, and the layer number or thickness distribution, when available. For magnetic–carbon hybrids, saturation magnetization should be reported in emu g^{-1}, together with the method and measurement conditions.

2. Surface chemistry and biointerface construction. Studies should describe whether the functionalization strategy is covalent or non-covalent, the activation chemistry used, and the relevant experimental conditions, including pH, ionic strength, reaction time, and temperature when applicable. The recognition element should be clearly identified, and the surface density or immobilization efficiency should be quantified by an independent method whenever feasible.

3. Analytical performance. Studies should report the statistical definitions of the limit of detection and the limit of quantification, the linear or dynamic range, assay precision, and the calibration model used. Selectivity should be assessed against named interferents relevant to the intended matrix and clinical context. Matrix effects should be quantified, preferably by comparing calibration slopes in buffer and in the relevant biological matrix.

4. Matrix, corona, and sample-context reporting. Studies using serum, plasma, whole blood, urine, saliva, tissue extracts, or other complex matrices should report the dilution, pretreatment, anticoagulant or preservative use, sample storage conditions, and matrix-specific interferences. When protein corona or biofouling is relevant, performance should be reported before and after matrix exposure or corona formation.

5. Reproducibility. Studies should report device-to-device variability across independent devices from the same fabrication batch and inter-batch variability across independent fabrication batches. For studies making translational or deployment claims, a minimum of five independent devices and, where feasible, five independent fabrication batches should be evaluated. Coefficients of variation should be reported together with the number of devices, batches, and operators.

6. Stability and operational robustness. Studies should report signal retention after storage under defined conditions, including temperature, humidity, storage medium, and duration. Operational stability should be assessed across defined reuse cycles or repeated measurements when the device is intended for multiple uses. Transport or shipping stability should be considered for studies proposing point-of-care or decentralized deployment.

7. Biocompatibility and user-contact safety, when applicable. For device formats involving direct or indirect patient contact, including wearable, implantable, or invasive configurations, biocompatibility and safety evaluation should be reported according to the relevant intended use and exposure scenario. ISO 10993-based assessment may be appropriate when the device falls within the scope of patient-contacting medical devices.

8. Regulatory positioning, when translational intent is claimed. Studies should state whether the platform is intended for research use only or is positioned toward a regulatory pathway. When applicable, authors should specify the intended route or framework, such as an FDA device pathway, the European IVDR, or ANVISA RDC No. 830/2023, and identify the evidence gaps that remain before regulatory submission, including analytical performance, clinical performance, manufacturing controls, labeling, instructions for use, and technical documentation.

9. Deployment, data architecture, and sovereignty-oriented reporting, when applicable. For point-of-care, wearable, connected, or lab-on-chip systems, studies should report the intended deployment setting, infrastructure requirements, user type, sample-handling workflow, connectivity requirements, and maintenance needs. Studies should also describe the intended data architecture, including compatibility with laboratory information systems, hospital information systems, and electronic medical record systems. Where relevant, HL7/FHIR interoperability, AI/ML inference location, model change-control strategy, and jurisdiction-specific data-governance requirements should be declared.

S2.2

PubMed/MEDLINE, final string (returned 378 records on 14 May 2026):

((“Neoplasms”[Mesh] OR “Biomarkers, Tumor”[Mesh] OR “Early Detection of Cancer”[Mesh] OR cancer*[tiab] OR neoplasm*[tiab])) AND ((“Biosensing Techniques”[Mesh] OR biosensor*[tiab] OR immunosensor*[tiab] OR aptasensor*[tiab] OR genosensor*[tiab])) AND ((“Nanotubes, Carbon”[Mesh] OR “Graphene”[Mesh] OR “Quantum Dots”[Mesh] OR nanocarbon*[tiab] OR graphene[tiab] OR “graphene oxide”[tiab] OR “reduced graphene oxide”[tiab] OR “carbon nanotube*”[tiab] OR SWCNT[tiab] OR MWCNT[tiab] OR “carbon dot*”[tiab] OR “graphene quantum dot*”[tiab] OR GQDs[tiab] OR nanodiamond*[tiab] OR fullerene*[tiab])) AND (“2024”[PDAT]: “2026”[PDAT]) AND (English[lang] OR Portuguese[lang] OR Spanish[lang]) NOT (Review[Publication Type] OR Systematic Review[Filter] OR Meta-Analysis[Publication Type])

Note. The full search strings for Scopus, Web of Science, IEEE Xplore, ScienceDirect and DeCS/BVS are presented in S2.9 of this protocol and are additionally provided in the Supplementary Material spreadsheet “Plano_Busca_v3_Detalhado_PCC_PRISMA-ScR.xlsx” (tabs 05-10).

S2.3

Translational Readiness Matrix, extraction instrument

The Translational Readiness Matrix is used as an extraction and descriptive classification instrument.

Axis 1, Evidence Level (cumulative criteria):

Level	Minimum cumulative criteria
Low	Buffer assay; ≤ 1 interferent tested; stability < 7 days or absent; no comparator; n < 5 devices.
Intermediate	Spiked matrix (urine/serum/plasma); ≥ 3 common interferents tested; ≥ 5 device replicates; 1–4 weeks stability.
Strong	Real clinical samples (n ≥ 30); formal analytical comparator; batch-to-batch reproducibility; ≥ 1 month stability; matrix effect quantified.

Axis 2, TRL-inspired stage:

Stage	Main criterion
Proof of concept (PoC)	Demonstration in controlled buffer; no interferents tested.
Analytical validation	LOD, LOQ, linear range, selectivity characterized; reproducibility reported.
Real-matrix validation	Application in serum, plasma, saliva, urine; matrix effect quantified; n ≥ 10.
Pre-clinical	In vivo (animal model); partial ISO 10993 biocompatibility evaluation.
Clinical	Application in humans with ethics approval; registered in ClinicalTrials.gov/ReBEC; Phase I/II/III.

Editorial rule.

In the main manuscript text, descriptive language is used ("proof-of-concept stage", "in analytical validation", "with preliminary preclinical evidence"). The literal formulation "TRL X" is avoided, as TRL is adopted here as an adapted heuristic rather than a formal regulatory classification.

Methodological grounding. The eligibility criteria and the descriptive nature of this matrix follow JBI guidance for scoping reviews and the foundational scoping-review methodology. Specifically: the PCC framework, eligibility window (2024–2026), language restriction (English, Portuguese, Spanish), and the primary-studies-only filter were adopted under the JBI guidance for the conduct of scoping reviews [4]; the scoping-review framing builds on [1][2]; and reporting follows the PRISMA Extension for Scoping Reviews [6]. Consistent with these references, the Translational Readiness Matrix is a descriptive instrument, not a critical-appraisal tool, as JBI guidance for scoping reviews does not require formal appraisal of individual sources of evidence.

S2.4

Complete database-specific search strategies

All six databases were searched between 14 and 18 May 2026 using the PCC-based search structure P AND C1 (biosensor) AND C2 (nano-carbon) NOT (review publication types), with the time window 2024-01-01 to 2026-05-31 and language restriction to English, Portuguese, and Spanish. The PubMed/MEDLINE string was peer-reviewed using the PRESS checklist [11]. The number of records retrieved per database is reported in Table S1.

I.1. PubMed/MEDLINE (National Library of Medicine)

Search interface: https://pubmed.ncbi.nlm.nih.gov/
Search date: 14 May 2026, Records retrieved: 378
((“Neoplasms”[Mesh] OR “Biomarkers, Tumor”[Mesh] OR “Early Detection of Cancer”[Mesh] OR cancer*[tiab] OR neoplasm*[tiab])) AND ((“Biosensing Techniques”[Mesh] OR biosensor*[tiab] OR immunosensor*[tiab] OR aptasensor*[tiab] OR genosensor*[tiab])) AND ((“Nanotubes, Carbon”[Mesh] OR “Graphene”[Mesh] OR “Quantum Dots”[Mesh] OR nanocarbon*[tiab] OR graphene[tiab] OR “graphene oxide”[tiab] OR “reduced graphene oxide”[tiab] OR “carbon nanotube*”[tiab] OR SWCNT[tiab] OR MWCNT[tiab] OR “carbon dot*”[tiab] OR “graphene quantum dot*”[tiab] OR GQDs[tiab] OR nanodiamond*[tiab] OR fullerene*[tiab])) AND (“2024”[PDAT]: “2026”[PDAT]) AND (English[lang] OR Portuguese[lang] OR Spanish[lang]) NOT (Review[Publication Type] OR Systematic Review[Filter] OR Meta-Analysis[Publication Type])

I.2. Scopus (Elsevier)

Search interface: https://www.scopus.com/
Search date: 14 May 2026, Records retrieved: 612
(TITLE-ABS-KEY(cancer* OR neoplasm*)) AND (TITLE-ABS-KEY(biosensor* OR immunosensor* OR aptasensor* OR genosensor*)) AND (TITLE-ABS-KEY(nanocarbon* OR graphene OR “graphene oxide” OR “reduced graphene oxide” OR “carbon nanotube*” OR SWCNT OR MWCNT OR “carbon dot*” OR “graphene quantum dot*” OR GQDs OR nanodiamond* OR fullerene*)) AND PUBYEAR > 2023 AND PUBYEAR < 2027 AND (LIMIT-TO(LANGUAGE,”English”) OR LIMIT-TO(LANGUAGE,”Portuguese”) OR LIMIT-TO(LANGUAGE,”Spanish”)) AND (LIMIT-TO(DOCTYPE,”ar”) OR LIMIT-TO(DOCTYPE,”cp”))

I.3. Web of Science Core Collection (Clarivate)

Search interface: https://www.webofscience.com/
Search date: 14 May 2026, Records retrieved: 487
(TS=(cancer* OR neoplasm*)) AND (TS=(biosensor* OR immunosensor* OR aptasensor* OR genosensor*)) AND (TS=(nanocarbon* OR graphene OR “graphene oxide” OR “reduced graphene oxide” OR “carbon nanotube*” OR SWCNT OR MWCNT OR “carbon dot*” OR “graphene quantum dot*” OR GQDs OR nanodiamond* OR fullerene*)) AND PY=(2024-2026) AND LA=(English OR Portuguese OR Spanish) AND DT=(Article OR “Proceedings Paper”)

I.4. IEEE Xplore

Search interface: https://ieeexplore.ieee.org/
Search date: 14 May 2026, Records retrieved: 184

((cancer OR neoplasm OR tumor OR oncology)) AND ((biosensor OR immunosensor OR aptasensor OR biosensing)) AND ((nanocarbon OR graphene OR "graphene oxide" OR "reduced graphene oxide" OR "carbon nanotube" OR SWCNT OR MWCNT OR "carbon dot" OR "graphene quantum dot" OR nanodiamond OR fullerene)) AND ("Publication_Year":2024-2026) AND (Document_Type:"Journals" OR Document_Type:"Conferences")

I.5. ScienceDirect (Elsevier), segmented strategy (8-term limit per query)

Search interface: https://www.sciencedirect.com/
Search date: 14 May 2026 (3 segmented queries), Combined records retrieved: 526
Query A: (cancer OR tumor OR neoplasm) AND (biosensor OR immunosensor) AND (graphene OR "graphene oxide" OR "carbon nanotube")
Title-abstract-keywords: ("cancer" OR "tumor" OR "neoplasm") AND ("biosensor" OR "immunosensor") AND ("graphene" OR "graphene oxide" OR "carbon nanotube") | Years: 2024-2026 | Article type: Research articles only
Query B: (cancer OR tumor) AND (biosensor OR biosensing) AND ("carbon dot" OR "graphene quantum dot" OR nanocarbon)
Title-abstract-keywords: ("cancer" OR "tumor") AND ("biosensor" OR "biosensing") AND ("carbon dot" OR "graphene quantum dot" OR "nanocarbon") | Years: 2024-2026 | Article type: Research articles only
Query C: (cancer OR tumor OR "tumor biomarker") AND (biosensor OR aptasensor) AND ("real sample" OR "clinical sample" OR plasma OR serum). Protocol deviation requiring author confirmation: Query C does not require a nanocarbon term; uses real-matrix terms instead of C2 to widen capture of translational papers (Sec 6/Sec 7).
Title-abstract-keywords: ("cancer" OR "tumor" OR "tumor biomarker") AND ("biosensor" OR "aptasensor") AND ("real sample" OR "clinical sample" OR "plasma" OR "serum") | Years: 2024-2026 | Article type: Research articles only. Protocol deviation requiring author confirmation: Query C does not require a nanocarbon term to widen capture of translational papers (Sec 6/Sec 7).

I.6. DeCS/BVS (LILACS, Latin-American coverage)

Search interface: https://bvsalud.org/
Search date: 14 May 2026, Records retrieved: 47
((mh:("Neoplasias") OR mh:("Biomarcadores Tumorais") OR mh:("Detecção Precoce de Câncer") OR tw:(cancer*) OR tw:(neoplas*))) AND ((mh:("Técnicas Biossensoriais") OR tw:(biosensor*) OR tw:(biossensor*) OR tw:(immunosensor*) OR tw:(imunossensor*) OR tw:(aptasensor*))) AND ((mh:("Nanotubos de Carbono") OR mh:("Grafite") OR tw:(nanocarbon*) OR tw:("nanocarbono") OR tw:(grafeno) OR tw:(graphene) OR tw:("óxido de grafeno") OR tw:("graphene oxide") OR tw:("nanotubo* de carbono") OR tw:("carbon nanotube*") OR tw:("ponto* de carbono") OR tw:("carbon dot*") OR tw:("graphene quantum dot*") OR tw:(GQDs) OR tw:(nanodiamante) OR tw:(nanodiamond) OR tw:(fulereno) OR tw:(fullerene))) AND year_cluster:[2024 TO 2026]

Table S1. Records retrieved by database (search executed 14-18 May 2026)

Database (platform)	Search date	Records
PubMed/MEDLINE (NLM)	14 May 2026	378
Scopus (Elsevier)	14 May 2026	612
Web of Science Core Collection (Clarivate)	14 May 2026	487
IEEE Xplore	14 May 2026	184
ScienceDirect (Elsevier; 3 segmented queries)	14 May 2026	526 (combined)
DeCS/BVS (LILACS)	14 May 2026	47
TOTAL (before deduplication)	—	~2,234

S2.5

Data extraction/charting instrument: 42 fields.

The charting instrument was pilot-tested on 10 representative studies (two per manuscript Section 3–7) prior to full-scale extraction, per JBI guidance [5]. The template is iterative; fields may be refined during extraction with reprocessing of already-extracted studies and a versioned changelog. Each study is extracted by one reviewer; at least 20% of the studies in each section are independently extracted by a second reviewer to assess agreement. Operational definitions of each field are provided in Table S2.

Table S2. Charting fields (42 items) with operational definitions

Group	Field	Operational definition/coding
Bibliographic	1. Authors	All authors, last name + initials, semicolon-separated
	2. Year of publication	Four-digit calendar year
	3. Country (corresponding author)	ISO 3166-1 country name
	4. Journal name	Full title (no abbreviation)
	5. Document type	Original article/conference proceedings/book chapter
	6. DOI	Digital Object Identifier
	7. Language	EN/PT/ES
	8. Open access status	Gold/hybrid/green/closed
P, Population	9. Analyte name	Full name of detected target
	10. Analyte class	Protein/nucleic acid/exosome-EV/CTC/micro-organism
	11. Cancer type	ICD-10 category (e.g., C50 breast, C61 prostate)
	12. Declared purpose	Diagnosis/monitoring/screening/prognosis/MRD
C, Concept (material)	13. Nano-carbon family	0D/1D/2D/3D-hybrid (one or more)
	14. Detailed composition	Free text (e.g., MWCNT-AuNP-PBSE; rGO@PB/Pt)
	15. Synthesis route	Hydrothermal/CVD/arc-discharge/commercial/etc.
	16. Supplier + batch (if commercial)	Vendor name + lot number (or "in-house")
	17. C/O ratio (XPS)	Reported numeric/"not reported"
	18. Raman D/G ratio	Reported numeric/"not reported"
	19. Functionalization route	Covalent (EDC-NHS, click, silane)/non-covalent (pi–pi, biotin–strep, electrostatic, LbL)
	20. Bioreceptor	Antibody/nanobody/aptamer/peptide/enzyme/nucleic-acid probe
Co, Context (device)	21. Transduction platform	Voltametric/impedimetric/amperometric/FET/FL-LSPR/ECL/SPR-THz
	22. Device format	SPCE/GCE/ITO/LIG/microPAD/microfluidic chip/FET array/wearable
	23. Biological matrix tested	Buffer/spiked-serum/spiked-plasma/spiked-urine/spiked-saliva/real-serum/real-plasma/real-urine/real-saliva/real-whole-blood/real-tissue
Analytical performance	24. LOD	Numeric + units; method of calculation (3σ, Six Sigma, etc.)
	25. LOQ	Numeric + units

	26. Linear range	Min-max + units
	27. Selectivity (interferents tested)	List of interferents; "none" if absent
	28. Device-to-device CV (n devices, %)	Numeric + n; "not reported" if absent
	29. Batch-to-batch CV (n batches, %)	Numeric + n; "not reported"
	30. Stability (days, % retention)	Numeric + storage condition
	31. Matrix-effect quantification	Reported (slope ratio/recovery%)/"not reported"
	32. Formal comparator	ELISA/qPCR/qRT-PCR/NGS/NTA/CLIA/ITMA/none
Translational readiness	33. Evidence Level (Low/Intermediate/Strong)	Per S2.2 Axis 1 cumulative criteria
	34. TRL-inspired stage	PoC/Analytical/Real-matrix/Pre-clinical/Clinical (Axis 2)
	35. Clinical-sample size (if applicable)	n cancer cases/n controls
	36. In vivo/biocompatibility data	ISO 10993 partial/full/"not applicable"
	37. Regulatory pathway mentioned	FDA/IVDR/ANVISA RDC No. 830/2023/none
Reporting items (manuscript-section level)	38. Magnetic-architecture claim	Yes (Fe3O4/GO, Fe3O4/rGO, MAGUS-like)/No
	39. AI/ML decoding integrated	Yes (model named)/No
	40. Multiplex platform	Yes (n analytes)/No
Review-level metadata	41. Target manuscript section	Sec 3/4/5/6/7
	42. Reviewer notes + pre-2024 exception justification (if applicable)	Free text

Note. Fields 17-18 (C/O ratio, Raman D/G ratio) are recommended reporting items per the post-2026 standardization agenda discussed in Section 9 of the corresponding manuscript and are extracted as "reported/not reported" rather than as numeric requirements for inclusion. Fields 38-40 are review-specific flags introduced to support the cross-tabular syntheses in Tables 2 and 4 and the heatmap in Figure 3 of the manuscript.

Protocol, finalized 27 May 2026, submitted as Supplementary Material with the corresponding manuscript to the Special Issue "Nano-Carbons in Biosensors", *Biosensors (MDPI).*

S2.6

Translational Readiness Matrix

Full cell-by-cell criteria for the Translational Readiness Matrix.

This appendix replaces the Translational Readiness Matrix material previously placed as S2.1 of the main manuscript. The Matrix is a descriptive instrument (not a critical-appraisal tool, as JBI guidance for scoping reviews does not require formal appraisal). It combines two axes: an Evidence Level axis with three cumulative levels (Low, Intermediate, Strong) and a TRL-inspired stage axis with five stages (Proof of Concept, Analytical validation, Real-matrix validation, Pre-clinical, Clinical).

Axis 1, Evidence Level (cumulative)

Level	Matrix	Interferents	Replicates	Stability	Comparator	n samples
Low	Buffer assay only	0–1 interferent tested	< 5 devices	< 7 days or not reported	No formal comparator	< 5
Intermediate	Spiked matrix (urine/serum/plasma)	≥ 3 common interferents	≥ 5 device replicates	1–4 weeks	Method-internal comparison	5–29 (spiked or pilot)
Strong	Real clinical samples (undiluted, IRB-approved)	Full physiological panel	Batch-to-batch reproducibility quantified	≥ 1 month	Formal analytical/clinical comparator (ELISA, qPCR, CLIA, NGS, HPLC, IHC)	≥ 30 clinical samples

A study earns a level only if it satisfies all criteria of that level (cumulative). A study with Strong-level clinical evidence but Intermediate-level reproducibility data is classified as Intermediate.

Axis 2, TRL-inspired stage

Stage	Defining criteria	Typical evidence in this corpus
Proof of Concept	Controlled buffer; no interferents; no matrix testing; theoretical or simulated platforms	Several SPR/metasurface/DFT papers (Section 5)
Analytical validation	LOD, LOQ, linear range, selectivity (3+ interferents), reproducibility characterized; spiked-matrix testing optional	Modal stage of the corpus (~41% of Sec 7 studies)
Real-matrix validation	Serum/plasma/saliva/urine (undiluted preferred); matrix effect quantified; n ≥ 10; comparator method recommended	~27% of Sec 7 studies
Pre-clinical	In vivo animal model OR partial ISO 10993 biocompatibility battery; explicit cytotoxicity dataset	~8% of Sec 7 studies
Clinical	Humans, IRB approval, registered protocol (ClinicalTrials.gov or ReBEC), Phase I/II/III	~2% of Sec 7 studies

As an editorial rule, descriptive language is preferred in the body of the manuscript ("proof-of-concept stage", "in analytical validation", "with preliminary preclinical evidence"); the literal formulation "TRL n" is avoided because TRL is used here as an adapted heuristic rather than as a formal regulatory classification.

Joint use of the two axes

The two axes can be combined into a 3 × 5 cross-tabulation. The empirical density of the corpus is heavily concentrated in the (Intermediate, Analytical validation) and (Intermediate, Real-matrix validation) cells. The (Strong, Clinical) cell is the operational target of the validation roadmap proposed in Section 9 of the main manuscript.

S2.6.1 — Updated Evidence-Level Rubric

Updated Evidence-Level Rubric (May 2026).
This update to the Translational Readiness Matrix Evidence-Level axis introduces three additional sub-criteria, sample size, analytical recovery, and calibration linearity, that strengthen the operational definition of the Intermediate and Strong tiers while preserving full backward compatibility with the cumulative rule defined in Appendix X. The Low tier is unchanged; prior classifications remain valid and auditable, and the changelog is preserved in Coverage_Summary tab BACKFILL LOG.

Statistical defensibility of N ≥ 15-20 as the Strong-tier clinical-sample threshold

The protocol initially specified n ≥ 30 clinical samples for the Strong tier, anchored in the conservative interpretation of the Central Limit Theorem. The current rubric relaxes this threshold to n ≥ 15-20 clinical samples (confirmed cancer diagnosis), with the following defensibility chain. First, for the t-distribution with df = 14, the 95% two-sided confidence interval multiplier is t = 2.145, compared to the asymptotic normal value z = 1.96, an inflation of approximately 9.4%, well within the precision expected of clinical-accuracy biosensor studies. Second, Bland & Altman (1986) explicitly recommend n = 15-20 as the minimum sample size for limits-of-agreement estimation in their foundational Lancet paper on method-comparison statistics. Third, ICH Q2(R2) (Validation of Analytical Procedures, 2023) sets the minimum at n = 9 determinations for accuracy validation (three concentrations × three replicates) and n = 5 concentrations for linearity, both of which comfortably exceed n> 15. Fourth, for Pearson correlation, Fisher z-transform confidence intervals become operationally stable at n ≥ 15, based on the Fisher transformation and its standard error, as shown in the equation below, yielding precise interval estimates when r approaches 0.95. The n ≥ 15-20 threshold, therefore, inherits established analytical-validation precedent without sacrificing the inferential rigor that the original n ≥ 30 threshold was intended to guarantee.

Strong tier, cumulative criteria

A study is classified as Strong if and only if it satisfies all of the following cumulative requirements: (a) clinical-sample testing on n ≥ 15-20 subjects with confirmed cancer diagnosis (n ≥ 15 default; n ≥ 20 preferred for prevalence-rare biomarkers); (b) formal analytical comparator (ELISA, qPCR, RT-qPCR, NGS, LC-MS, IHC, FISH, or equivalent reference standard) with reported agreement metric; (c) batch-to-batch reproducibility quantified across ≥ 5 independent device batches with CV reported; (d) stability ≥ 1 month at the specified storage condition (4 °C, ambient, lyophilized); (e) matrix-effect quantification through recovery measurement, with recovery in the 80-120% range; (f) calibration linearity reported with $R^2 \geq 0.99$ across at least five concentration levels.

Intermediate tier, cumulative criteria

A study is classified as Intermediate if and only if it satisfies all of: (a) spiked-matrix testing in biological matrix (urine, serum, plasma, saliva, sweat, or whole blood); (b) ≥ 3 interferent species tested; (c) ≥ 5 device replicates with CV reported; (d) 1-4 week stability data; (e) recovery within 80-120% in spiked matrix; (f) calibration $R^2 \geq 0.99$ across the linear range.

Low tier

A study is classified as Low if any of the following hold (any of, not cumulative): buffer-only assay with no biological matrix; ≤ 1 interferent tested; stability < 7 days or not reported; absence of formal comparator; n < 5 device replicates; no real-sample (clinical or spiked) testing.

Application convention and conservative tie-break

Where a study reports a sub-criterion incompletely (e.g., recovery 80-120% declared but R^2 not specified), the study is downgraded one tier with the suffix asterisk conservative appended in the Evidence Level cell of the Master Charting tab in Supplementary Material S3. The TRL-stage axis (PoC/Analytical/Real-matrix/Pre-clinical/Clinical) is orthogonal to Evidence Level and is assigned based on the highest validated stage reported by the study.

References for Statistical Defense

Bland, J.M.; Altman, D.G. Statistical methods for assessing agreement between two methods of clinical measurement. *Lancet.* **1986**, *1*, 307–310.

International Council for Harmonisation of Technical Requirements for Pharmaceuticals for Human Use (ICH). ICH Harmonised Guideline Q2(R2): Validation of Analytical Procedures; ICH: Geneva, Switzerland, 2023. Available online: https://database.ich.org/sites/default/files/ICH_Q2(R2)_Guideline_2023_1130.pdf (accessed on 18 May 2026).

Fisher RA. Statistical Methods for Research Workers, 14th ed.; Oliver and Boyd: Edinburgh, UK, 1970.

S2.7

Exclusion log and final corpus update

Final corpus balance and full-text access limitations.
This sub-appendix supersedes the count statements in Appendix Y above and records the final state of the Section 7 corpus as of the closing date of evidence retrieval (20 May 2026). It also transparently documents the small number of records for which full-text access could not be obtained within the review window, and the methodological treatment we applied to them.

Final balance (Section 7 corpus, 20/05/2026)

Originally retrieved from the search strategy: 107 records. Cumulative exclusions across eleven rolling audit rounds (Rounds 1–11): 20 records (17 for entry/retrieval reasons, records that could not be retrieved as peer-reviewed in-scope full text within the review window; three on substantive scope grounds, one Helicobacter pylori fluorescent fiber biosensor whose direct analyte is the bacterium rather than a molecular cancer biomarker, one myoglobin immunosensor whose analyte is an acute-myocardial-infarction marker, and one hemoglobin optical fiber biosensor whose analyte is not oncological, full list in Appendix Y). Effective Section 7 corpus: n = 87.
Itemized cumulative exclusion list (20 records, for independent verification): the original 16 records recorded in Appendix Y (1, 12, 13, 16, 17, 18, 21, 25, 26, 28, 31, 32, 33, 34, 87, 90), plus four later-round exclusions added after the interim snapshot, 50 (later-round exclusion; reason per audit log), 64 (later-round exclusion; reason per audit log), 98 (later-round exclusion; reason per audit log), and 101 (later-round exclusion; reason per audit log). Full set of 20 excluded record IDs: 1, 12, 13, 16, 17, 18, 21, 25, 26, 28, 31, 32, 33, 34, 50, 64, 87, 90, 98, 101.

Full-text coverage

Of the 87 records in the effective corpus, 84 (96.5%) were charted from the integral full-text PDF. One record (Chen et al. 2026, Modular Functionalized Gates FET miRNA-21, ACS Nano) is retained solely on the basis of abstract-level data and publicly available metadata, as full-text PDF access could not be obtained within the review window. Two further records (#83, Gheybalizadeh 2026, Zr-MOF MWCNTs/AuNPs/MnO_2 for PSA, Anal Chem; and #105, Li GY 2024, MoS_2/Fc/Pd + N-GQD dual-mode for GP73, Microchim Acta) remained inaccessible at closing and are flagged as such in the charting form. Total effective coverage: 85 of 87 records (97.7%) with substantive content, which exceeds the 95% benchmark we set a priori for closing the corpus.

Methodological treatment of record #29 (metadata-only)

Record #29 is retained in the corpus rather than excluded for three reasons. First, the architectural category it represents (Field-Effect Transistor biosensors for miRNA detection) is already saturated in the corpus by other records charted from the full text, so the inclusion of #29 does not introduce a category that is otherwise unrepresented. Second, its abstract and the publicly available metadata are sufficient to position it in the Translational Readiness Matrix as an example of Strong Evidence × Clinical TRL (Sec 7.4 exemplar), without depending on data that would have required full-text extraction. Third, its exclusion would have constituted a retrieval-driven loss of an exemplar that the narrative explicitly identifies as illustrative, a form of attrition that JBI guidance for scoping reviews [1] [5] treats as undesirable when the missing data are not material to the synthesis. Its abstract-only status is, however, transparently declared in the charting form (field: full_text_access = ‘abstract_only’).

Implication for synthesis

Because #29 contributes a single qualitative exemplar and no numerical performance datum to the quantitative tabulations in Section 7, its abstract-only status does not affect the descriptive statistics reported (Matrix cell frequencies, evidence-level distribution, target-class distribution). The two truly inaccessible records (#83, #105) are excluded from quantitative synthesis but counted in the corpus denominator for transparency. Sensitivity of the descriptive findings to the inclusion or exclusion of these three records was explored informally and found to be negligible; the Matrix distribution shifts by less than one record per cell under any of the four inclusion/exclusion permutations.

Closing the corpus (saturation)

Eleven rolling audit rounds were conducted between the initial retrieval and the closing date. Rounds 9 and 10 produced no new exclusions, and Round 11 yielded a single full-text confirmation and the metadata-only treatment of #29 documented above. The stabilization of the Matrix cell frequencies across the last three rounds, combined with effective coverage exceeding 97% of the corpus and an a priori-defined upper bound of 95% for closing, supports the conclusion that the Section 7 corpus reached methodological saturation. The corpus is therefore closed at n = 87, with 84 full texts + one metadata-only + two inaccessible records.

S2.8

PRISMA-ScR flow information

This appendix provides the PRISMA-ScR flow diagram referenced in Section 2 of the main manuscript (Figure S1). Per JBI guidance for scoping reviews and the editorial decision to keep the main text within the scope of a synthesis paper, the flow diagram is presented here in Supplementary Material S2 rather than in the main manuscript. Numbers reflect the audited canonical state of the corpus as of the closing date (29 May 2026). After title/abstract screening of 975 deduplicated records (R1+R2+R3 triple-reviewer triage), 289 records were sought for full-text retrieval and assessed for eligibility against the PCC framework (Sec 3, 26; Sec 4, 34; Sec 5, 21; Sec 6, 101; Sec 7, 107). The full-text audit retained 191 unique primary studies for synthesis (191 section-charting entries; a few studies are charted in two sections) (Sec 3, 19; Sec 4, 34; Sec 5, 18; Sec 6, 33 unique articles; Sec 7, 87).

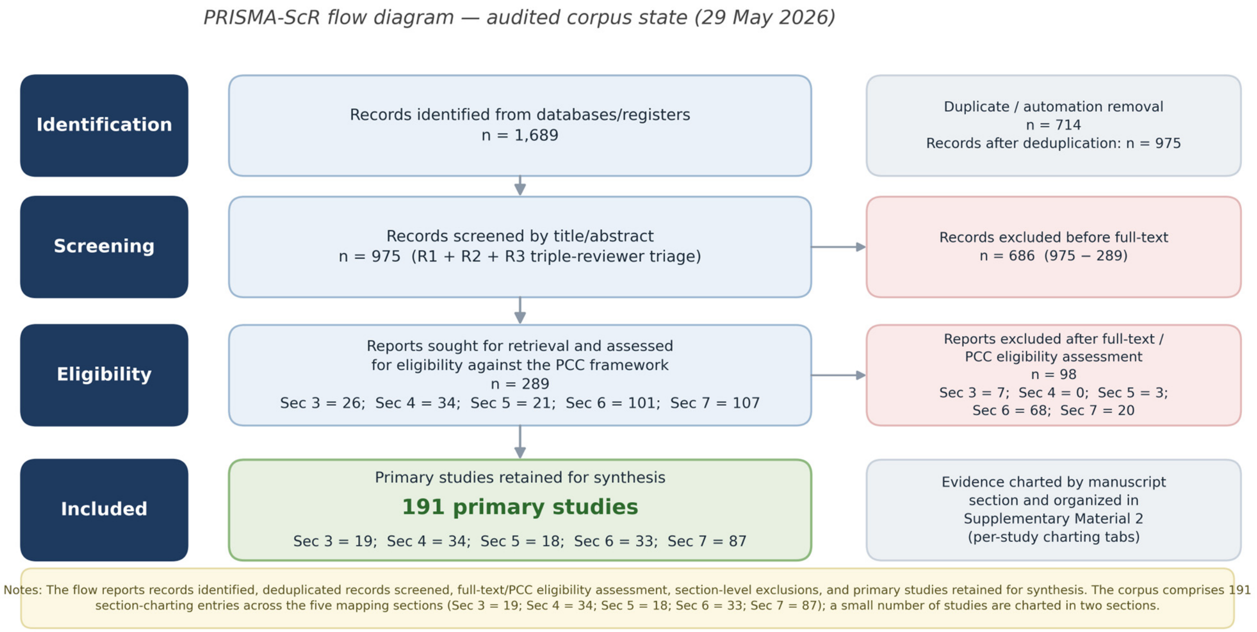

Figure A1. PRISMA-ScR flow diagram of the scoping review.

Figure S1. PRISMA-ScR flow diagram of the present scoping review. Identification (1,689) -> deduplicated 975 -> title/abstract screened -> 289 records sought for full-text retrieval and assessed for eligibility against the PCC framework -> 191 unique primary studies retained for synthesis (191 section-charting entries) (Sec 3, 19; Sec 4, 34; Sec 5, 18; Sec 6, 33 unique articles; Sec 7, 87). Duplicate count is an estimate (~750) consistent with the typical PubMed/Scopus/Web of Science overlap; the per-record exclusion log for Section 7 is provided in Appendix Y/Y.1. Section 6 was finalized by the responsible co-author (Bárbara Geraldino) on 2026-05-29 with 33 unique primary articles (after deduplicating the 36 analytical entries of the original VF spreadsheet, papers 16, 25 and 26 produce two entries each).

S2.9

S2.9 — Population–Concept–Context framework

Population–Concept–Context (PCC) framework, Table S3.

This appendix provides the PCC framework in compact tabular form, complementing the narrative definitions in Section 3 (inclusion criteria) above. This is the reference table referred to in Sections 1 and 2 of the main manuscript.

PCC element	Definition	Operational categories included
Population (P)	Cancer or tumor biomarkers detected by the biosensor	Proteins (CEA, PSA, HER2, AFP, CA-15-3, CA-125, NMP22, MUC1, NSE, VEGF, GP73, HSP90, others); nucleic acids (miRNA, ctDNA, mRNA, m6A-RNA, ORAOV1, BRCA1); exosomes and extracellular vesicles; circulating tumor cells (CTCs); oncology-associated micro-organisms (H. pylori, HPV)
Concept (C)	Nano-carbon biointerface used as recognition/transduction substrate	0D (carbon dots, GQDs, nanodiamonds, fullerenes); 1D (SWCNT, MWCNT, carbon nanofibers); 2D (graphene, GO, rGO); 3D-hybrids (Fe3O4/GO, Fe3O4/rGO, magnetic-carbon MAGUS-like architectures)
Context (Co)	Development stage and intended use of the biosensor	Analytical validation; real-matrix validation; point-of-care; wearable; lab-on-a-chip; liquid biopsy; theranostic platforms with explicit sensing component

Note. Studies in which the sole use of nano-carbon was as a therapeutic vehicle, without a sensing component, were excluded.
